# Tailoring Mesalazine Nanosuspension Using Chitosan Polyelectrolyte Complexes with Alginate and Alginate/Hydroxypropyl-Methylcellulose Phthalate

**DOI:** 10.3390/pharmaceutics16121489

**Published:** 2024-11-21

**Authors:** Amélia Aparecida Rocca Pereira, José Vitor Melchiades Aparecida, Maria Eduarda Ramalho, Leonardo Miziara Barboza Ferreira, Maria Palmira Daflon Gremião

**Affiliations:** Faculty of Pharmaceutical Science, UNESP—São Paulo State University, Rodovia Araraquara-Jaú, Km 01, Araraquara 14801-902, Brazil; amelia.rocca@unesp.br (A.A.R.P.); jvm.aparecida@unesp.br (J.V.M.A.);

**Keywords:** chitosan, alginate, HPMC, nanosuspension, mesalazine

## Abstract

**Background/Objectives**: This study evaluated how the relative proportion of chitosan (CS) to the polyanions alginate (ALG) and hydroxypropyl-methylcellulose phthalate (HP) affects the colloidal properties of mesalazine (MSZ) nanosuspensions as a strategy to produce particles with specific characteristics. **Methods**: Nanosuspensions were prepared using a bottom–up approach based on acid–base reactions and were modified with CS in a binary mixture with ALG or a ternary mixture with ALG and HP. The particle size, polydispersity index (PDI), zeta potential, morphology, and drug association efficiency were analyzed. **Results**: Higher proportions of CS relative to the polyanions resulted in smaller, less polydisperse particles. The zeta potential inversion was influenced by the relative proportion of CS in the system. These results were consistent over 30 days and pH exerted an influence on the magnitude of the observed effect. The optimized NS modified with binary CS/ALG blends had the following properties at pH 6.0: an average particle size of 324.9 nm, PDI of 0.5, and zeta potential of +40.8 mV; at pH 4.0, it had an average particle size of 310.4 nm, PDI of 0.4, and zeta potential of +43.6 mV. The optimized NS modified with ternary CS/ALG/HP had the following properties at pH 6.0: an average particle size of 316.7 nm, PDI of 0.5, and zeta potential of +33.9 mV; at pH 4.0, it had an average particle size of 363.5 nm, PDI of 0.6, and zeta potential of +33.9 mV. **Conclusions**: CS-based polyelectrolyte complexes with ALG and ALG/HP offer an approach to modulating the properties of MSZ nanosuspensions, enabling the production of particles with tailored characteristics.

## 1. Introduction

In recent years, there has been exciting advancement in enhancing the therapeutic efficacy of drugs through the use of drug delivery systems (DDSs). When a drug is incorporated into these systems, the properties governing its release, interaction with the biological environment, and stability are no longer solely dependent on its physicochemical characteristics but can be modulated by the DDS. Incorporating drugs into a DDS can enhance stability, reduce pre-systemic metabolism, increase the drug residence time at the target site, and enable targeting to specific sites. This approach supports dose reductions, more appropriate dosing regimens, fewer adverse effects, and improved patient adherence to therapy [1].

Several intestinal diseases require personalized treatment tailored to specific pathophysiological conditions, with typical examples including inflammatory bowel diseases (IBDs) and colorectal cancer (CRC) [2,3]. In this context, nanotechnology-based DDSs offer a valuable strategy to meet these personalized treatment needs. Nanometer-scale particles can alter the retention time in the colon, modulating permeability effects and epithelial retention through endocytosis by immune cells. They also help prevent rapid elimination of the drug from the system, which is often an issue in IBD-associated diarrhea [4,5,6]. Additionally, studies have shown that nanoparticle (250 nm) and microparticle (3.0 µm) transport through inflamed colonic mucosa is particle-size dependent: microparticles tend to remain in the superficial mucosal layers, while nanoparticles penetrate deeper.

Mesalazine (MSZ) is a drug classified as BCS Class IV, characterized by a low solubility and low permeability, resulting in variable bioavailability. Drugs in this category pose significant challenges for drug delivery scientists. To address these issues, nanosuspensions (NSs) have been explored to enhance dissolution rates and, consequently, improve oral bioavailability. NSs are submicron colloidal dispersions of drug particles stabilized by surfactants, polymers, or a combination of both. They can be produced through chemical precipitation (bottom–up) or by disintegration processes (top–down) in a liquid dispersion medium. In the bottom–up technique, drug particles are molecularly dispersed in a solvent and precipitated through the addition of an antisolvent [7]. For drugs that are weak acids or bases with pH-dependent solubility, precipitation through acid–base neutralization reactions is particularly effective [8].

Nanosuspensions are an attractive tool to address the problems presented by insoluble or poorly soluble drugs in water as well as needs of personalization demands [9]. However, NS particles may not have adequate properties to overcome the physiological barriers of the administration routes or the hostile biological environment, impairing the effectiveness of NSs. Functionalization of the NS surface is possible and represents a valuable strategy to promote targeted delivery, generating innovative drug delivery systems with personalized properties. The coating of the NS can occur through electrostatic interactions, dipole–dipole interactions, hydrogen bonds, or hydrophobic interactions [10,11]. Thus, some structural characteristics of the polymers and the nanosuspension must be considered when developing these systems.

Among the various polymers, the polycation chitosan (CS) is particularly notable for its ability to form polyelectrolyte complexes with different types of polyanions. The strategy of polyelectrolyte complexation serves as an important alternative for modulating interactions with mucus [12]. In this study, alginate (ALG) and hydroxypropylmethylcellulose phthalate (HP), both polyanions, were selected due to their advantageous properties for intestinal applications and colonic drug delivery systems [1,13]. This research systematically developed MSZ nanosuspensions coated with multifunctional layers composed of CS, ALG, and/or HP. The impact of the synthesis variables, such as polymer proportion and composition, on the resulting particle properties was thoroughly investigated.

## 2. Materials and Methods

We purchased the following materials: low-molecular-weight chitosan (Sigma Aldrich, St. Louis, MI, USA, Cat. No. 448869), sodium alginate (Sigma Aldrich, W201502), hydroxypropylmethylcellulose phthalate (Shin Etsu Chemical, Tokyo, Japan), glacial acetic acid (Qhemis, São Paulo, Brazil), sodium hydroxide (Vetec, Odense, Denmark), and other analytical-grade reagents from commercial suppliers. Mesalazine was a donation of Laboratórios Ferring (São Paulo, Brazil). Milli-Q ultrapure water (Millipore, Burlington, MA, USA) was used for sample preparation.

### 2.1. Evaluation of Mesalazine Solubility at Different pH Values

MSZ solubility was determined at pH levels of 4 and 6. To prepare the samples, an excess amount of MSZ was added to ensure medium saturation. The samples were then agitated at room temperature using a tube homogenizer for 24 h and subsequently centrifuged at 3000 rpm for 15 min. The supernatant was filtered (Millipore Amicon UFC201024, 10 KDa, Burlington, MA, USA), and the samples were analyzed using a UV–VIS spectrophotometer Cary 60 (Agilent Technologies, CA, USA).

### 2.2. Preparation and Characterization of MSZ Nanosuspensions by Bottom–Up Process

An MSZ stock solution (10 mg/mL) was prepared by adding HCl (1 mol/L) to dissolve the drug, with the solution kept under magnetic stirring until complete dissolution. Subsequently, four different concentrations (10, 5, 3.3, and 1.66 mg/mL) were prepared, with the pH adjusted to 4 and 6. To precipitate the nanosuspensions, a NaOH solution (1 mol/L) was added dropwise to the MSZ solution under magnetic stirring for 15 min, resulting in precipitate formation. For the preparation of the molecularly dispersed drug, MSZ (0.8 mg/mL) was dissolved in water, and the pH values were adjusted to 4 and 6 using NaOH (1 mol/L) and HCl (1 mol/L).

#### 2.2.1. Determination of the Percentage of Precipitated Particles

To evaluate the percentage of precipitated particles, the samples containing four different concentrations of MSZ (10, 5, 3.3, and 1.66 mg/mL) were centrifuged at 3000 rpm for 15 min, and the supernatant was filtered (Amicon UFC201024, 10 kDa). MSZ quantification was performed using a UV–VIS spectrophotometer with a previously validated analytical calibration curve. The results were calculated using the following equation:

AE = (Total MSZ mass − free MSZ mass in the supernatant)/(Total MSZ mass)

#### 2.2.2. Size, Polydispersity Index, and Zeta Potential Analyses of Mesalazine Nanosuspensions

The mean hydrodynamic diameter was analyzed using dynamic light scattering (DLS) at 25 °C, with a detection angle of 173°, using a Zetasizer Nano ZS (Malvern Instruments, Malvern, UK). A 7:3 *v*/*v* dilution was used, and the samples were analyzed in triplicate. The zeta potential was determined through electrophoretic mobility, also using the Zetasizer Nano ZS (Malvern Instruments). For this analysis, the samples were prepared in triplicate. The results are expressed as the median and were used in a comparative study of the nanosuspensions and the molecularly dispersed drug, as well as the empty polymeric systems and the systems with MSZ added under both preparation conditions.

#### 2.2.3. Evaluation of the Redispersibility of Nanosuspensions

To evaluate the redispersibility of the NSs, the samples were left to stand for 24 h, and then homogenized, and the formation of compact sediment at the bottom of the flask was observed.

### 2.3. Preparation and Characterization of MSZ Nanosuspensions Associated with Polyelectrolytes

#### 2.3.1. Preparation of Polymeric Dispersions

CS, ALG, and HP polymers were dispersed at a concentration of 5 mg/mL. CS was dispersed in 0.1 mol/L acetic acid, ALG in ultra-purified water, and HPMC in a 1 mol/L NAOH solution with mechanical stirring for approximately 30 min.

#### 2.3.2. Preparation of CS/ALG and CS/ALG/HP Systems Without the Drug

The nanostructured systems were prepared using the technique of polyelectrolyte complexation, where the cationic polymer (CS) was combined with the anionic polymers (ALG and HP). For the system composed of CS and ALG, the CS dispersion was slowly added to ALG using a 1 mL graduated pipette, with magnetic stirring maintained for 15 min. For the system containing HP, a similar procedure was followed, with the gastro-resistant polymer added to the preformed dispersion while maintaining stirring for 15 min.

#### 2.3.3. Development Systems with Addition of Solubilized Drug and in Nanosuspension Containing Polymeric Blend of CS/ALG and CS/ALG/HPMC

For the incorporation of the solubilized MSZ into the polyelectrolyte complexes, solutions of MSZ (0.8 mg/mL) were prepared and mixed with the ALG solution. This mixture was then slowly added to the CS solution under magnetic stirring for 15 min. For the ternary formulations, the same steps were followed, with HP being added to the preparations, followed by pH adjustment to 4 and 6. The formulations were stored in a refrigerator and protected from light.

#### 2.3.4. Preparation of Binary and Ternary Polymeric Systems with the Drug in Nanosuspension

To prepare the MSZ nanosuspension within polymeric systems, the drug was first prepared at a concentration of 1.66 mg/mL and then added to the ALG solution. The mixture was then dripped (using a 1 mL syringe) into the CS dispersion, under magnetic stirring for 15 min. The same procedure was followed for the ternary system, with HP being added after the preformed preparation. The formulations were then stored in a refrigerator, protected from light. The samples were labeled with the initial prefix of each polymer (CS/ALG/HP) and their respective proportions (2:8, 8:2, 2:4:4, and 8:1:1). For the samples containing the drug in nanosuspension, the acronym (NS) was used as a suffix, while for the samples with the molecularly dispersed drug, the acronym (M) was used.

#### 2.3.5. Size, Polydispersity Index, and Zeta Potential Analyses of Mesalazine Nanosuspensions and Binary and Ternary Systems Without the Drug and with the Drug in Nanosuspension and Molecularly Dispersed

The average diameter and polydispersity index (PDI) of the particulate systems were determined using dynamic light scattering (DLS) and a Zetasizer Nano ZS at 25 °C, under a detection angle of 173°. The Z-average diameter was calculated based on the translational diffusion coefficient, which is influenced not only by the particle size but also by the concentration and ions present in the medium. The PDI was assessed, with values of 0.1 indicating a monodisperse system, values between 0.1 and 0.4 indicating moderate polydispersity, and values greater than 0.5 indicating high polydispersity. PDI values close to zero suggest the formation of a more homogeneous system, while values greater than 0.5 indicate a heterogeneous system with varying particle sizes [14]. The zeta potential was used to assess the physical stability of the formulations, as it reflects the surface charge of particles and the electrostatic repulsion between positive and negative charges.

#### 2.3.6. Evaluation of the Association Efficiency of Mesalazine in Molecular and Nanosuspension Forms to Polymeric Systems

To evaluate the association efficiency, MSZ was determined using an indirect method, which involved measuring the amount of free MSZ in the supernatant. For this, the samples were centrifuged at 3000 rpm for 30 min at 25 °C. The amount of free MSZ in the supernatant was calculated using the analytical curve obtained during method validation. Association efficiency (AE) was calculated using the following equation: AE = (MA − MQ)/MA × 100, where MA represents the total mass of MSZ added and MQ represents the mass of MSZ quantified in the supernatant.

#### 2.3.7. Morphological Analysis

The morphology of the raw material particles (MSZ), the nanosuspensions (NSs), and the formed complexes was analyzed using high-resolution scanning electron microscopy (SEM-FEG) and a JEOL JSM-7500 instrument. The samples were diluted 1:20 (*v*/*v*) in a 0.5% (*v*/*v*) Tween 20 dispersion to prevent particle agglomeration during the drying process. The samples were then fixed directly onto aluminum stubs using double-sided adhesive tape and left in a desiccator for 48 h. For imaging, the samples were coated with a conductive material (carbon), and photomicrographs were taken at a magnification of 30,000×.

## 3. Results and Discussion

Nanosuspensions (NSs) are colloidal dispersions of submicron particles containing solid drug particles smaller than 1 μm, and they can address many of the drug formulation and delivery challenges that are typically associated with poorly water- or lipid-soluble drugs [9,15,16]. In this study, MSZ nanosuspensions were prepared and coated with hydrophilic polymers. These modifications were intended to modulate biological responses by altering the surface characteristics of the nanosuspension particles.

### 3.1. Development of MSZ Nanosuspensions

**Figure 1:**
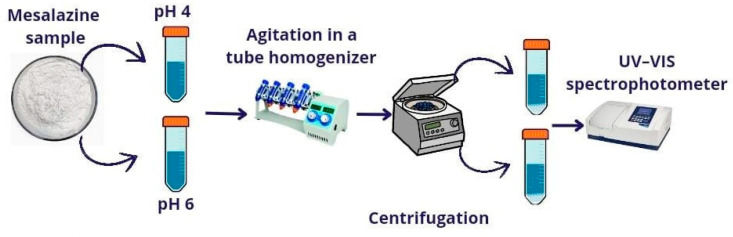
Schematic of MSZ nanosuspension preparation at pH 4 and pH 6.

Two methods can be used to obtain nanosuspensions (NSs): top–down and bottom–up methods. The top–down method involves breaking larger particles, using techniques such as milling, microfluidization, and high-pressure homogenization. However, this method requires significant time and energy inputs and may lead to the formation of amorphous particles or drug degradation. The bottom–up method, or chemical precipitation, involves preparing a saturated solution of the drug and precipitating it through acid–base titration. This method was employed in this work (Figure 1) [17,18,19]. The first step was to determine the concentration of MSZ to prepare the NS. The solubility of MSZ at pH 4 and pH 6 was found to be 0.88 mg/mL and 0.92 mg/mL, respectively. To prepare MSZ nanosuspensions using the bottom–up method, the drug was solubilized in an acid (HCl) and precipitated with NaOH at pH 4 or 6. Four different concentrations of MSZ were tested (10, 5, 3.3, and 1.66 mg/mL). The samples were evaluated for size, PDI, and zeta potential. Most of the tested samples showed negative zeta potential values and high PDI values, indicating the presence of a heterogeneous system. The concentration of 1.66 mg/mL MSZ resulted in the smallest Z-average diameter and acceptable PDI values at both pH levels (Table 1). Therefore, the concentration of MSZ selected for further study was 1.66 mg/mL, as it produced nanoscale particles.

The samples containing 1.66 mg/mL of MSZ, prepared at pH 4 and pH 6, were evaluated for the percentage of particle formation. The analyses were performed on the day of preparation, after 24 h, and after 168 h. The results showed that for the samples prepared at pH 4, 24.5% of the drug was in particulate form on the day of preparation. After 24 h, this percentage increased to 61.6%, and after 168 h, no further variation was observed. It is suggested that, due to the zwitterionic form of MSZ at this pH, both the amino and carboxylic groups are ionized and continue to interact. At pH 6, particle precipitation reached 48%. At this pH, the molecule is in its anionic form, with only the carboxylic groups ionized, which likely results in less molecular interaction and consequently, less particle formation. After 24 h and 168 h, no significant changes were observed, with the percentage of particles remaining between 44% and 45% (Table 2).

### 3.2. Nanosuspension Coated with CS/ALG or CS/ALG/HP Polymeric Blends

Many poorly soluble drugs, due to their high crystalline energy, have low solubility in water. Nanosuspensions (NSs) have been developed to address the challenges posed by drugs with high crystalline energy, which makes them insoluble in both lipids and aqueous vehicles. The solid state of NSs provides high weight-to-volume loading, making them ideal for localized delivery, where the administration volume is restricted, and high drug doses are required. The reduced particle size results in a high surface area, which increases the dissolution rate, thereby overcoming solubility-limited bioavailability. These types of drug delivery systems (DDSs) are often stabilized with surfactants and/or polymers [15,20]. In this work, we explored polymer blends (CS and CS/ALG) that, in addition to preventing particle agglomeration, provide mucoadhesion and gastroresistant properties to the formulations.

**Figure 2:**
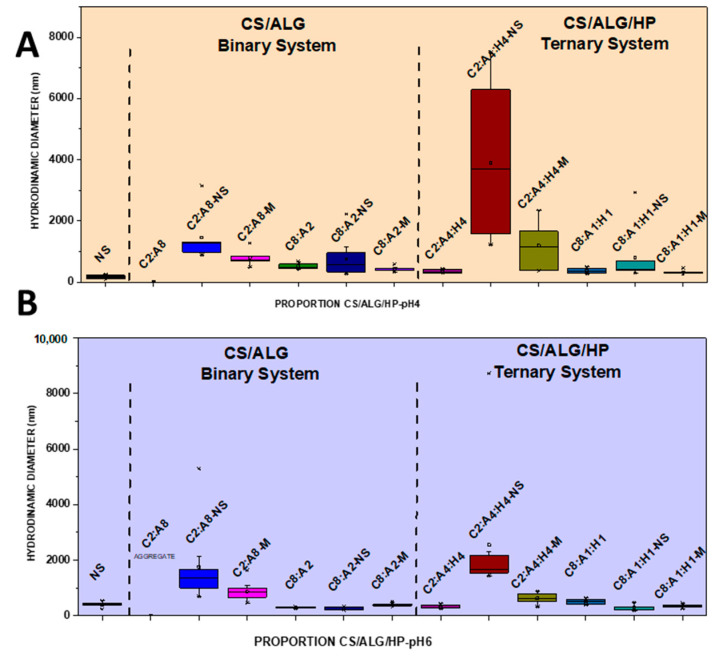
Boxplot summary for hydrodynamic diameter of CS/ALG and CS/ALG/HP particles at (**A**) pH 4 and (**B**) pH 6.

**Figure 3:**
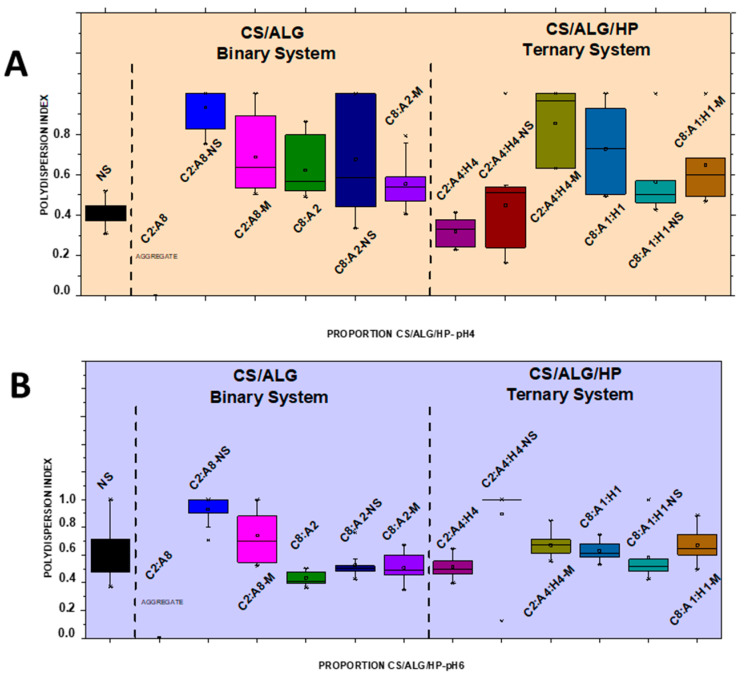
Boxplot summary for polydispersity index of CS/ALG and CS/ALG/HP particles at (**A**) pH 4 and (**B**) pH 6.

**Figure 4:**
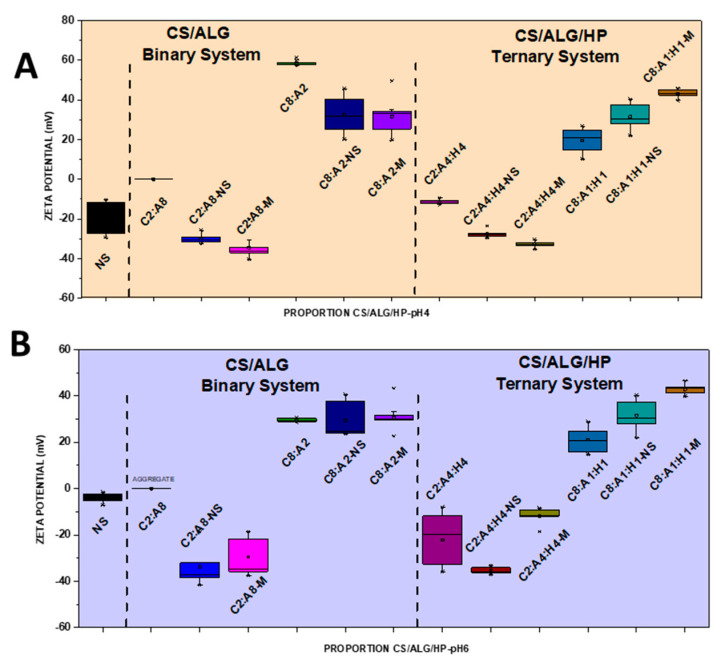
Boxplot summary for zeta potential of CS/ALG and CS/ALG/HP particles at (**A**) pH 4 and (**B**) pH 6.

NSs containing 1.66 mg/mL of MSZ at pH 4 and pH 6 were prepared. At pH 4, the NS showed a median diameter of 140.9 nm (Figure 2A), a PDI of 0.4 (Figure 3A), and a negative zeta potential of −19.6 mV (Figure 4A). These NSs were added to the ALG dispersions, and then the CS dispersion was gradually added. Various polymer proportions were tested. The NSs with higher proportions of CS exhibited smaller particle sizes, while those with lower CS proportions showed larger particle sizes. This suggests that the higher proportion of CS positive charges enhanced electrostatic interactions, promoting the formation of smaller particles. The NSs with a lower proportion of CS in the CS polymer blend exhibited high PDI values, indicating particle heterogeneity. The zeta potential values were positive in the samples with the highest proportion of CS, and the same behavior was observed in the ternary system with the drug in its molecularly dispersed form. For the zeta potential (Figure 4A), all samples, whether with the drug in an NS or molecularly dispersed, showed adequate electrical stability.

At pH 6, the data also indicated that the samples with higher proportions of CS exhibited a favorable mean diameter and zeta potential. However, it is important to note that such initial formulation screening trials may not provide accurate size measurement results. When the PDI is too high, the curve fit in the program is poor, which leads to non-convergence between repeated measurements and measurement errors. This observation is likely related to the molecular weight and degree of deacetylation of CS, which are crucial characteristics that influence the properties of polyelectrolytes, making them difficult systems to evaluate using light scattering. Previous studies have shown that the interaction between CS and ALG, when using low-molecular-weight CS, results in a reduction in particle size [21]. The samples prepared at pH 6 had a lower mean diameter (219.8 nm) compared to those prepared at pH 4, a PDI of 0.4 (Figure 2B and Figure 3B), and a zeta potential of −22.4 mV (Figure 4B). At pH 4, the samples with a lower proportion of CS showed particle sizes on the micrometer scale and heterodispersity when the drug was incorporated into the NS.

It was verified that after the addition of the drug, both in the form of an NS and molecularly dispersed, the smallest particle diameters were obtained in the binary (C8:A2) and ternary (C8:A1:H1) systems at pH 6, that is, in the systems with a higher ratio of CS in relation to the ALG and HP polymers. The negative charges of MSZ likely interacted electrostatically with the positive ionizable groups of CS, promoting a rearrangement of the polymer chains into a more compact network and resulting in smaller particles.

In a comparative study between binary and ternary systems, we observed that the C8:A2 samples (at pH 4) with the highest CS ratio in the system showed zeta potential values around +32 mV for both molecularly dispersed MSZ and MSZ in an NS. At pH 6, the zeta potential was in the +30.6 mV to 25 mV range, which does not represent a large variation due to the state of the drug dispersion (molecular vs. NS). This can be attributed to the proximity of the pH value to the pKa value of CS, due to the deprotonation of the amino groups in the molecule [21]. In contrast, the C8:A1:H1 samples, with the highest CS ratio in the system, showed zeta potential values of +43.3 mV and +30.7 mV for molecularly dispersed and NS MSZ, respectively. This occurred irrespective of the pH level.

### 3.3. Stability in Binary and Ternary Systems

**Figure 5:**
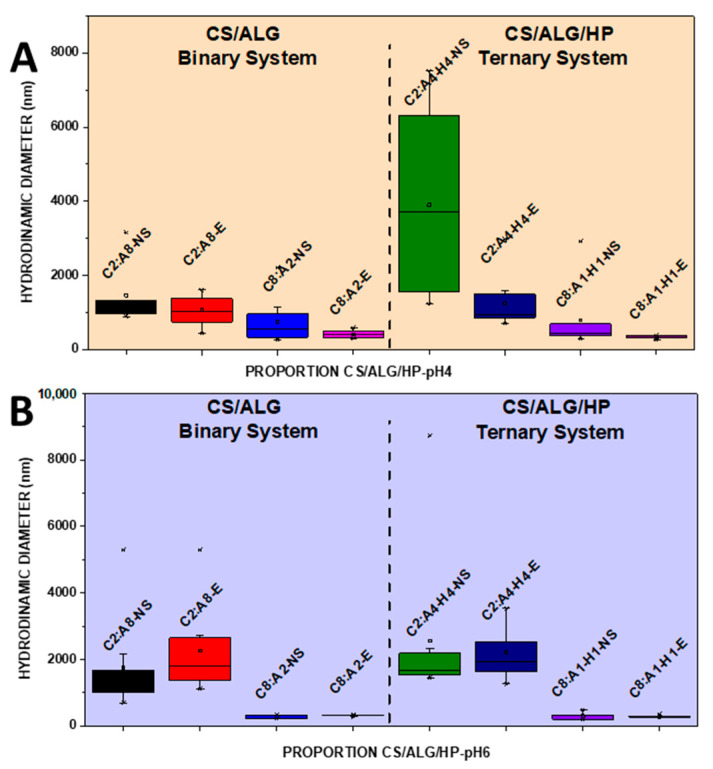
Boxplot summary for stability studies of CS/ALG and CS/ALG/HP. Hydrodynamic diameter measured at (**A**) pH 4 and (**B**) pH 6.

**Figure 6:**
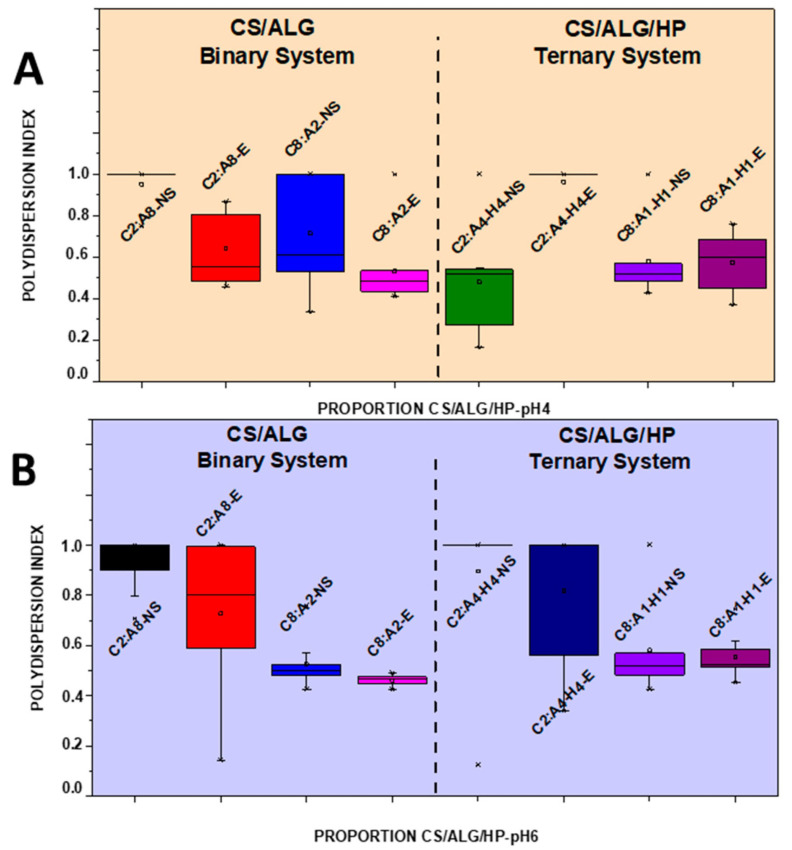
Boxplot summary for stability studies of CS/ALG and CS/ALG/HP. Polydispersity index measured at (**A**) pH 4 and (**B**) pH 6.

**Figure 7:**
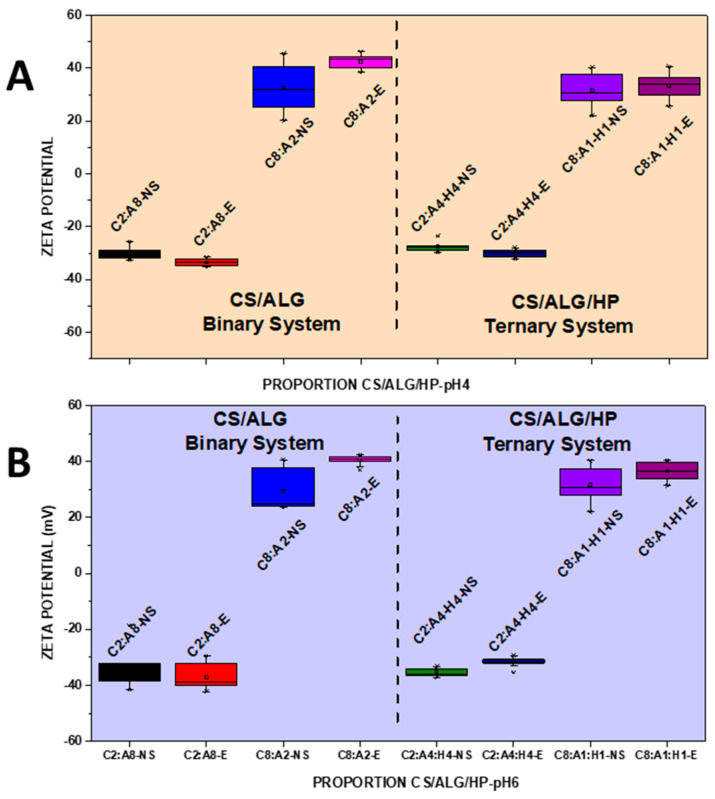
Boxplot summary for stability studies of CS/ALG and CS/ALG/HP. Zeta potential measured at (**A**) pH 4 and (**B**) pH 6.

Stability studies were carried out to verify if there was any changes in the particle diameter, as well as in the PDI and zeta potential of the binary and ternary polymeric systems with the drug added in an NS, over time. The samples are indicated as the initial prefix of each polymer (CH/ALG/HP), followed by their respective ratio (2:8, 8:2, 2:4:4, 8:1:1). For the samples analyzed before the 30-day period, the suffix NS was used and for the samples that were analyzed after 30 days, the suffix E was used. At pH 4, the ternary samples showed a reduction in the hydrodynamic diameter (Figure 5A) of the particles, without any changes in the PDI (Figure 6A) and the zeta potential (Figure 7A). In the binary system with a higher proportion of CS, there was a reduction in the particle diameter, as well as in the PDI since the zeta potential showed a significant increase over 30 days.

At pH 6, there was a slight increase in the hydrodynamic diameter and zeta potential of the binary and ternary samples containing a higher proportion of CS (Figure 5B and Figure 7B). The PDI remained stable (Figure 6B). The samples containing a lower proportion of CS showed quite similar behaviors, with an increase in particle size after 30 days. The PDI remained high and slight variations in zeta potential were observed.

### 3.4. Comparison of Size Values (Intensity and Number) of Binary and Ternary Systems

**Figure 8:**
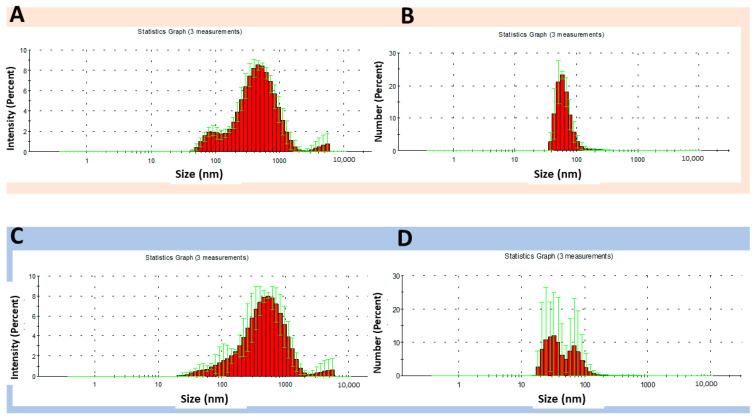
Size distribution of binary system C8:A2-NS at pH 4, expressed as the intensity (**A**,**B**) number of particles; (**C**) intensity and (**D**) number of particles at pH 6.

**Figure 9:**
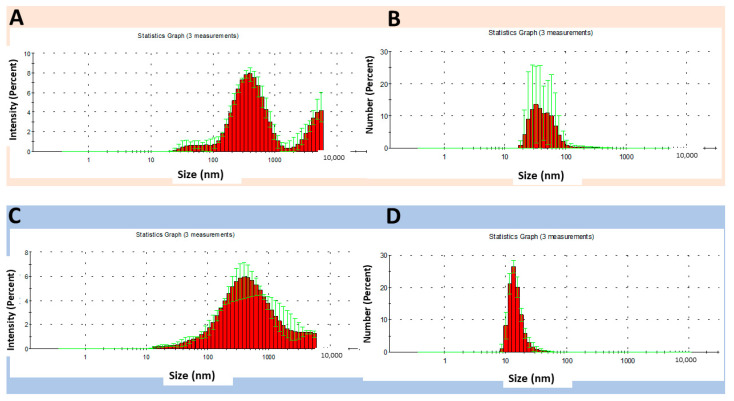
Size distribution of the ternary system C8:A1:H1-NS at pH 4, expressed as intensity (**A**,**B**) number of particles; (**C**) intensity and (**D**) number of particles at pH 6.

To refine our understanding of the properties of the most promising binary and ternary systems, we compared the size distributions in terms of intensity and number (Figure 8 and Figure 9). The two modes of measurement use distinct weighting factors. The intensity mode represents how much light is scattered by particles of different sizes, which can bias the measurement toward larger particles since they scatter much more light than smaller ones. On the other hand, the number mode represents the actual number of particles in each size range. The particles contribute equally to the distribution, reducing the impact of larger particles.

Among the binary systems, C8:A2-NS at pH 4 (Figure 8A) showed a multimodal distribution, with particles in the ranges of 40 to around 1200 nm. The Z-average value was 310.4 nm ± 9.3 and the PDI was 0.4. At pH 6, a slight distribution tail to the left was observed and the Z-average value was 324.9 nm ± 8.2 (Figure 8C). By analyzing the distribution of the number of particles at pH 4, an almost symmetric histogram was obtained, with higher percentages of particles in the range of 43 nm to 120 nm (Figure 8B). At pH 6, a bimodal distribution and peak broadening were observed (Figure 8D). The differences observed in the two measurement modes reflect the polydisperse nature of the system. Polyelectrolyte complexes are prone to aggregate formation, which can be observed in intensity measurements, as larger particles contribute disproportionately to the intensity distribution, overshadowing smaller particles.

For the ternary system C8:A1:H1-NS (Figure 9), the mean intensity percent values were distinct from the number percent values at both evaluated pH levels. The broadening of the intensity distribution histogram with increasing pH level suggests that the ionization behavior of polyelectrolytes can modify the interaction pattern of the NS and the formation of aggregates that can distort the size measurements. Comparing the Z-average values between the pH levels, it was observed that C8:A1:H1-NS had a particle size of 363.5 nm ± 29.3 and PDI of 0.6 at pH 4, and a particle size of 316.7 nm ± 34 and PDI of 0.5 at pH 6.0. In contrast, the narrowing of the number distribution histogram with increasing pH indicates the formation of a more homogeneous system.

### 3.5. Evaluation of the Association Efficiency of Solubilized Mesalazine and Nanosuspensions Incorporated in Binary (CS/ALG) and Ternary (CS/ALG/HP) Polymeric Systems

To evaluate the association efficiency (AE%) of MSZ in the binary and ternary polymeric systems, the sample with 1.66 mg/mL of MSZ, which had the smallest z-mean particle diameter and PDI, was selected for analysis, along with samples containing the drug in its molecularly dispersed form. The AE% of MSZ in the nanoparticles is shown in Table 3. The AE% for MSZ in the NS ranged from 43.2% to 61.7%, which is consistent with the results obtained for the precipitated particles, which ranged from 39.6% to 64.9%. Although higher association efficiencies and drug loadings have been reported in the literature, the obtained values for the MSZ in the NS were satisfactory compared to the molecularly dispersed MSZ [22]. For the molecularly dispersed MSZ formulations, the AE% ranged from 28.3% to 30.6%. The AE% values for the systems containing the NS were similar for both the binary and ternary systems. Samples containing a higher proportion of CS at pH 4 showed a higher association efficiency percentage. In contrast, the incorporation of the solubilized drug resulted in a lower percentage of particles adhering to the systems, as fewer drug molecules were available to interact with the functional groups of the polymers.

### 3.6. Morphological Analysis of Mesalazine and the Binary (CS/ALG) and Ternary (CS/ALG/HP) Polymeric Systems

**Figure 10:**
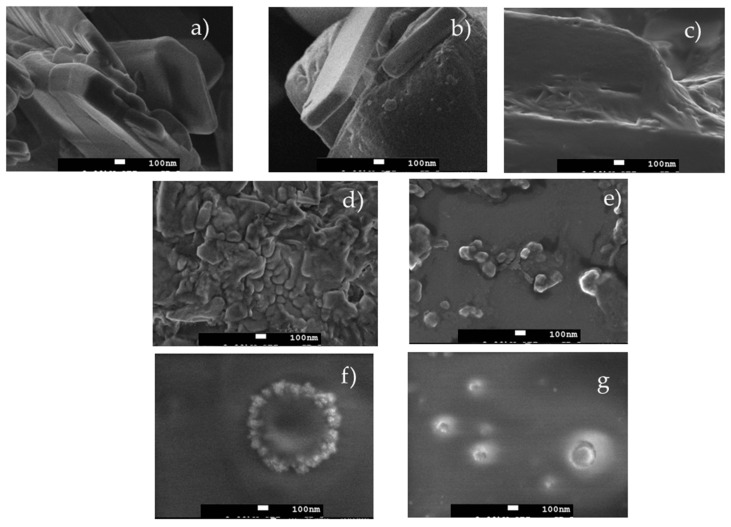
Analysis of the morphology using scanning electronic microscopy with 30,000× magnification. (**a**) MSZ powder, (**b**) MSZ-NS pH 4, (**c**) MSZ-NS pH 6, (**d**) CS/ALG + NS at pH 4, (**e**) CS/ALG + NS at pH 6, (**f**), CS/ALG/HPMC + NS ternary system at pH 4, (**g**), CS/ALG/HPMC + NS ternary system at pH 6.

The morphology of the MSZ in the raw material (commercially acquired), nanosuspension, and systems with the drug added at pH 4 and 6 were evaluated at a magnification of 10,000 times. Figure 10a shows that the MSZ in the raw material and the nanosuspension at pH 6 (Figure 10b) exhibited tabular crystals with a rough surface, while the nanosuspension at pH 4 (Figure 10c) presented tabular crystals with a smoother surface. The photomicrographs in Figure 10d,e reveal that the binary systems had an irregular round shape with the presence of agglomerates. In the ternary system, spherical-shaped particles were observed (Figure 10f,g), which, upon incorporation of the drug into a nanosuspension, exhibited a single-layer granular surface. Santos (2015) observed similar results with methotrexate samples, where the addition of HP as a steric stabilizer led to the formation of spherical-shaped particles, suggesting that the presence of HP is necessary for the formation the coating on the particle surface [7].

## 4. Conclusions

MSZ nanosuspensions were obtained using the bottom–up method based on acid–base reactions, with particle formation occurring under drug precipitation conditions. Suspensions in the nanoscale were obtained in a very restricted range (below 1.66 mg/mL of MSZ). The other investigated concentrations were not suitable for accurate DLS measurements and further surface modifications. Subsequently, the selected NSs were modified with polymeric systems of CS, ALG, and HP in different proportions using the polyelectrolyte complexation technique, avoiding the use of organic solvents and high amounts of energy. All the system tested showed highly polydispersity, which was evidenced by significant differences between the results from the intensity and number analysis modes for the size distribution. By comparing the systems with the same qualitative compositions, that is, comparing binary systems with each other and ternary systems with each other, it was observed that increasing the proportion of CS reduced the particle size, reduced or maintained the PDI, and promoted charge inversion of negatively charged particles to positively charged particles. These observations occurred regardless of the MSZ dispersion state (molecularly dispersed or in an NS) and were somehow independent of pH, which exerted a modulating effect on the intensity of the variation. In contrast, the direction of the effect of increasing CS on the association efficiency of the NS depended on the pH, since there was an increase in association efficiency at pH 4 and a decrease at pH 6.0. We suggest some probable mechanisms through which pH modulates the effect of increasing CS in NS samples of binary and ternary systems:

1. The magnitude of the size reduction in the NS was due to the increase in the CS proportion (at pH 4: ~1238 nm to 500 nm and 3720 to 424 nm for the binary and ternary systems, respectively; at pH 6.0: ~1358 nm to 276 nm and 1686 to 219 for the binary and ternary systems, respectively).
2. The magnitude of the reduction in PDI of the NS was due to the increase in the CS proportion (at pH 4: ~0.9 to 0.6 for the binary system; at pH 6.0: ~0.9 to 0.5 and 1.0 to 0.5 for the binary and ternary systems, respectively).
3. The charge inversion of the NS was due to the increase in the CS proportion (at pH 4: ~−30.8 mV to +31.9 mV and −28.4 mV to + 30.7 mV for the binary and ternary systems, respectively; at pH 6.0: ~−37.4 mV to + 24.6 mV and −36.2 to +30.6 for the binary and ternary systems, respectively).
4. At pH 4, increasing CS proportions in the NS slightly increased the AE%: ~57.4% to 61.7% and ~56.2% to 59.5% for the binary and ternary systems, respectively. At pH 6.0, there was a decrease in the AE%: ~57.3% to 43.4% and ~50.9% to 43% for the binary and ternary systems, respectively.

In summary, CS-based polyelectrolyte complexes with ALG and ALG/HP offer a promising approach to modulating the properties of MSZ nanosuspensions, allowing for the production of particles with tailored characteristics. The proportion of CS relative to the polyanions significantly influences key colloidal properties such as particle size and zeta potential. The pH value further modulates these effects, affecting the degree of property enhancement or reduction. Formulations with a higher CS content achieved spherical structures, nanoscale particle diameters, favorable size distributions, and electrical stability, indicating their suitability for potential technological applications. This effect was particularly notable in systems containing HP, where the photomicrographs revealed coating formation on the particle surfaces. Previous studies have demonstrated the feasibility of using polyelectrolyte complexation for mucoadhesion modulation in intestinal disease treatments employing DDSs with drugs like bevacizumab and camptothecin [11,12,13]. Although promising, CS-based polyelectrolyte complexation strategies face quality control and regulatory challenges in the pharmaceutical field [23]. When this type of strategy is used to modify the surface properties of preformed particles, a series of questions arise. It is important to highlight that these systems have a complex composition, as they combine the drug MSZ and high-molecular-weight polymers. Therefore, it is plausible to hypothesize that particles with different natures can co-exist in the formulation, for example, nanocomplexes containing only polymers, NSs without a coating, and coated NSs. Future studies should be concerned with understanding these details of system formation with an emphasis on deciphering how an elevated PDI can distort size and zeta potential measurements. Moreover, investigations of possible nanotoxicological effects using biological and biorelevant models are of utmost importance to ensure the safety and efficacy of these formulations in practical applications.

## Figures and Tables

**Table 1:** Evaluation of size, PDI, and zeta potential of MSZ nanosuspensions at pH 4 and pH 6.

pH	Concentration (mg/mL)	Size (nm)	PDI	Zeta Potential (mV)
4	1.66	366.2 ± 27.3	0.4 ± 0.2	−8.00 ± 2.9
6	1.66	390.8 ± 11.2	0.6 ± 0.2	−1.9 ±0.42

Size measurements are expressed as intensity distributions.

**Table 2:** Percentage of precipitated particles as a function of preparation time.

Sample	Time (h)	Percentage of MSZ in Precipitate (%)
NS pH 4	0	24.5
	24	61.6
	168	67.1
NS pH 6	0	48
	24	44
	168	45.1

**Table 3:** Evaluation of MSZ (molecularly dispersed or in nanosuspension) association efficiency with binary and ternary systems.

pH of Preparation	Association Efficiency (%)	
	MSZ	
	Solubilized MSZ (0.8 m/mL)	NS (1.66 mg/mL)
	Without Polyelectrolytes	
4	-	65.1
6	-	44
	**B** **inary system: CS/ALG (ratio 2:8)**	
4	30.5	57.4
6	29.5	57.3
	**B** **inary system: CS/ALG (ratio 8:2)**	
4	28.3	61.7
6	29.5	43.4
	**T** **ernary system: CS/ALG/HP (2:4:4)**	
4	30.6	56.2
6	28.3	50.9
	**T** **ernary system: CS/ALG/HP (8:1:1)**	
4	29.1	59.5
6	28.4	43.3

## Data Availability

The original contributions presented in the study are included in the article/Supplementary Materials. The data presented in this study are available on request from the corresponding author.

## Supplementary Materials

The following supporting information can be downloaded at: https://www.mdpi.com/article/10.3390/pharmaceutics16121489/s1, Figure S1. MSZ Nanosuspensions 1.66 mg/mL pH 4.0 (Intensity distribution), Figure S2: MSZ Nanosuspensions 1.66 mg/mL pH 4.0 (Number Distribution), Figure S3: MSZ Nanosuspensions 1.66 mg/mL pH 4.0 (Zeta Potential Distribution), Figure S4: MSZ Nanosuspensions 1.66 mg/mL pH 6.0 (Intensity distribution), Figure S5: MSZ Nanosuspensions 1.66 mg/mL pH 6.0 (Number distribution), Figure S6: MSZ Nanosuspensions 1.66 mg/mL pH 6.0 (Zeta Potential distribution), Figure S7: C8:A2-NS MSZ 1.66 mg/mL pH 4.0 (Intensity distribution), Figure S8: C8:A2-NS MSZ 1.66 mg/mL pH 4.0 (Number distribution), Figure S9: C8:A2-NS MSZ 1.66 mg/mL pH 4.0 (Zeta Potential distribution), Figure S10: C8:A2-NS MSZ 1.66 mg/mL pH 6.0 (Intensity distribution), Figure S11: C8:A2-NS MSZ 1.66 mg/mL pH 6.0 (Number distribution), Figure S12: C8:A2-NS MSZ 1.66 mg/mL pH 6.0 (Zeta Potential distribution), Figure S13: C8:A1:H1-NS MSZ 1.66 mg/mL pH 4.0 (Intensity distribution), Figure S14: C8:A1:H1-NS MSZ 1.66 mg/mL pH 4.0 (Number distribution), Figure S15: C8:A1-H1-NS MSZ 1.66 mg/mL pH 4.0 (Zeta Potential distribution), Figure S16: C8:A1:H1-NS MSZ 1.66 mg/mL pH 6.0 (Intensity distribution), Figure S17: C8:A1:H1-NS MSZ 1.66 mg/mL pH 6.0 (Number distribution), Figure S18: C8:A1-H1-NS MSZ 1.66 mg/mL pH 6.0 (Zeta Potential distribution).
