# Supplementary material for: Tailoring Mesalazine Nanosuspension Using Chitosan Polyelectrolyte Complexes with Alginate and Alginate/Hydroxypropyl-Methylcellulose Phthalate
Source: Pharmaceutics. 2024 Nov 21;16(12):1489. doi: 10.3390/pharmaceutics16121489 (PMC11676705; doi:10.3390/pharmaceutics16121489)
Supplement: Supplementary file 1 [file pharmaceutics-16-01489-s001.zip › ! Supplementary Material.pdf]

Supplementary Material

Size d.nm	Mean Intensity Percent	Std Dev Intensity Percent
0,4000	0,0	0,0
0,4632	0,0	0,0
0,5365	0,0	0,0
0,6213	0,0	0,0
0,7195	0,0	0,0
0,8332	0,0	0,0
0,9649	0,0	0,0
1,117	0,0	0,0
1,294	0,0	0,0
1,499	0,0	0,0
1,736	0,0	0,0
2,010	0,0	0,0
2,328	0,0	0,0
2,696	0,0	0,0
3,122	0,0	0,0
3,615	0,0	0,0
4,187	0,0	0,0
4,849	0,0	0,0

Size d.nm	Mean Intensity Percent	Std Dev Intensity Percent
5,615	0,0	0,0
6,503	0,0	0,0
7,531	0,0	0,0
8,721	0,0	0,0
10,10	0,0	0,0
11,70	0,0	0,0
13,54	0,0	0,0
15,69	0,0	0,0
18,17	0,0	0,0
21,04	0,0	0,0
24,36	0,1	0,1
28,21	0,1	0,2
32,67	0,1	0,2
37,84	0,2	0,1
43,82	0,3	0,3
50,75	0,5	0,4
58,77	0,6	0,4
68,06	0,7	0,2

Size d.nm	Mean Intensity Percent	Std Dev Intensity Percent
78,82	0,9	0,3
91,28	1,1	0,8
105,7	1,7	1,2
122,4	2,5	1,5
141,8	3,8	1,9
164,2	5,3	2,3
190,1	7,0	2,9
220,2	8,5	3,4
255,0	9,6	3,4
295,3	10,0	2,9
342,0	9,7	1,9
396,1	8,6	0,9
458,7	7,0	1,7
531,2	5,2	2,6
615,1	3,5	2,7
712,4	2,2	2,0
825,0	1,2	1,1
955,4	0,5	0,5

Size d.nm	Mean Intensity Percent	Std Dev Intensity Percent
1106	0,1	0,1
1281	0,0	0,0
1484	0,0	0,0
1718	0,0	0,0
1990	0,0	0,0
2305	0,0	0,0
2669	0,0	0,0
3091	0,1	0,2
3580	0,5	0,6
4145	1,4	1,3
4801	2,6	2,3
5560	4,3	2,5
6439	0,0	0,0
7456	0,0	0,0
8635	0,0	0,0
1,000e4	0,0	0,0

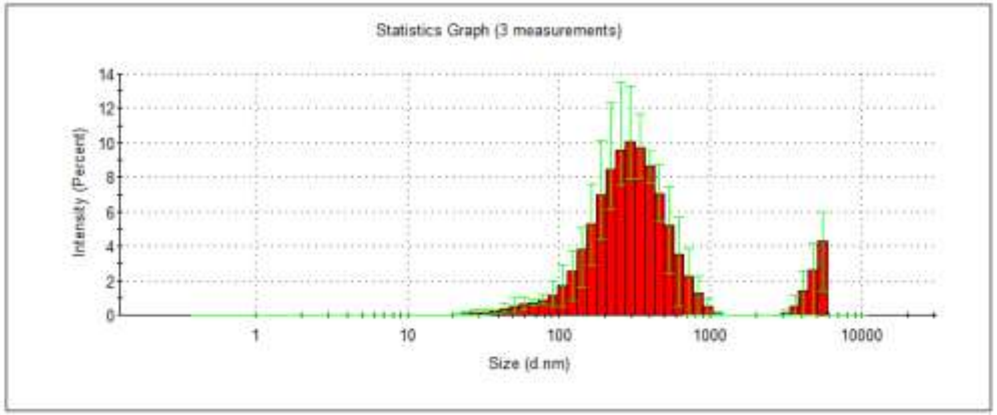

Figure S1. MSZ Nanosuspensions 1.66 mg/mL pH 4.0 (Intensity distribution)

Size d.nm	Mean Number Percent	Std Dev Number Percent
0,4000	0,0	0,0
0,4632	0,0	0,0
0,5365	0,0	0,0
0,6213	0,0	0,0
0,7195	0,0	0,0
0,8332	0,0	0,0
0,9649	0,0	0,0
1,117	0,0	0,0
1,294	0,0	0,0
1,499	0,0	0,0
1,736	0,0	0,0
2,010	0,0	0,0
2,328	0,0	0,0
2,696	0,0	0,0
3,122	0,0	0,0
3,615	0,0	0,0
4,187	0,0	0,0
4,849	0,0	0,0

Size d.nm	Mean Number Percent	Std Dev Number Percent
5,615	0,0	0,0
6,503	0,0	0,0
7,531	0,0	0,0
8,721	0,0	0,0
10,10	0,0	0,0
11,70	0,0	0,0
13,54	0,0	0,0
15,69	0,0	0,0
18,17	2,3	3,9
21,04	7,5	12,9
24,36	10,0	17,3
28,21	8,4	12,3
32,67	9,5	4,7
37,84	14,8	10,4
43,82	17,1	13,8
50,75	13,3	11,4
58,77	7,7	6,9
68,06	3,7	3,4

Size d.nm	Mean Number Percent	Std Dev Number Percent
78,82	1,7	1,6
91,28	0,9	1,0
105,7	0,6	0,8
122,4	0,5	0,8
141,8	0,5	0,7
164,2	0,4	0,6
190,1	0,4	0,5
220,2	0,3	0,4
255,0	0,2	0,3
295,3	0,1	0,2
342,0	0,1	0,1
396,1	0,1	0,1
458,7	0,0	0,0
531,2	0,0	0,0
615,1	0,0	0,0
712,4	0,0	0,0
825,0	0,0	0,0
955,4	0,0	0,0

Size d.nm	Mean Number Percent	Std Dev Number Percent
1106	0,0	0,0
1281	0,0	0,0
1484	0,0	0,0
1718	0,0	0,0
1990	0,0	0,0
2305	0,0	0,0
2669	0,0	0,0
3091	0,0	0,0
3580	0,0	0,0
4145	0,0	0,0
4801	0,0	0,0
5560	0,0	0,0
6439	0,0	0,0
7456	0,0	0,0
8635	0,0	0,0
1,000e4	0,0	0,0

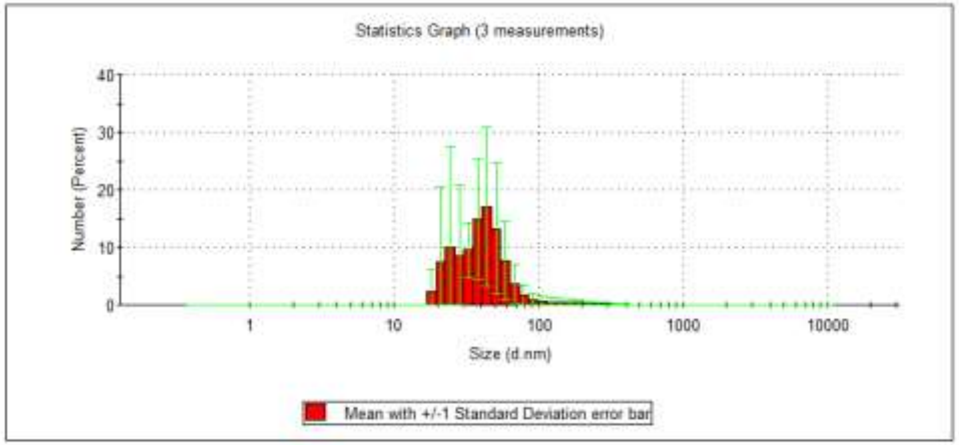

Figure S2: MSZ Nanosuspensions 1.66 mg/mL pH 4.0 (Number Distribution)

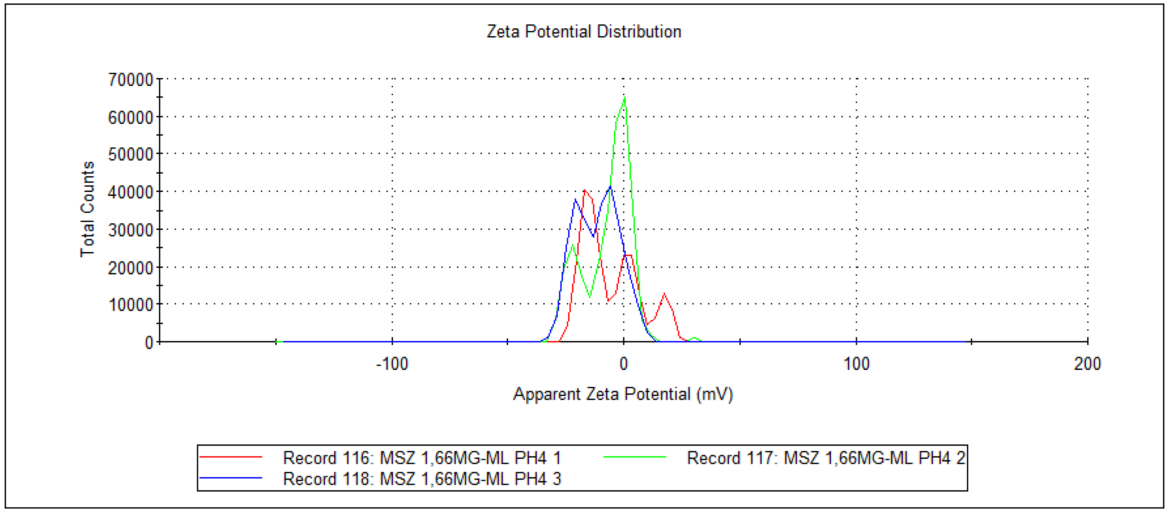

Figure S3: MSZ Nanosuspensions 1.66 mg/mL pH 4.0 (Zeta Potential Distribution)

Size d.nm	Mean Intensity Percent	Std Dev Intensity Percent
0,4000	0,0	0,0
0,4632	0,0	0,0
0,5365	0,0	0,0
0,6213	0,0	0,0
0,7195	0,0	0,0
0,8332	0,0	0,0
0,9649	0,0	0,0
1,117	0,0	0,0
1,294	0,0	0,0
1,499	0,0	0,0
1,736	0,0	0,0
2,010	0,0	0,0
2,328	0,0	0,0
2,696	0,0	0,0
3,122	0,0	0,0
3,615	0,0	0,0
4,187	0,0	0,0
4,849	0,0	0,0

Size d.nm	Mean Intensity Percent	Std Dev Intensity Percent
5,615	0,0	0,0
6,503	0,0	0,0
7,531	0,0	0,0
8,721	0,0	0,0
10,10	0,0	0,0
11,70	0,0	0,0
13,54	0,0	0,0
15,69	0,0	0,0
18,17	0,0	0,0
21,04	0,0	0,0
24,36	0,0	0,0
28,21	0,0	0,0
32,67	0,0	0,0
37,84	0,0	0,0
43,82	0,0	0,0
50,75	0,0	0,0
58,77	0,1	0,1
68,06	0,2	0,1

Size d.nm	Mean Intensity Percent	Std Dev Intensity Percent
78,82	0,6	0,2
91,28	1,0	0,4
105,7	1,6	0,5
122,4	2,2	0,6
141,8	2,8	0,6
164,2	3,3	0,7
190,1	3,8	0,8
220,2	4,2	0,8
255,0	4,5	0,8
295,3	4,8	0,8
342,0	4,9	0,7
396,1	5,0	0,7
458,7	5,0	0,6
531,2	4,9	0,5
615,1	4,8	0,4
712,4	4,7	0,3
825,0	4,5	0,3
955,4	4,3	0,3

Size d.nm	Mean Intensity Percent	Std Dev Intensity Percent
1106	4,1	0,4
1281	3,9	0,5
1484	3,6	0,5
1718	3,4	0,6
1990	3,2	0,7
2305	2,9	0,7
2669	2,7	0,7
3091	2,4	0,7
3580	2,1	0,7
4145	1,9	0,6
4801	1,6	0,6
5560	1,3	0,5
6439	0,0	0,0
7456	0,0	0,0
8635	0,0	0,0
1,000e4	0,0	0,0

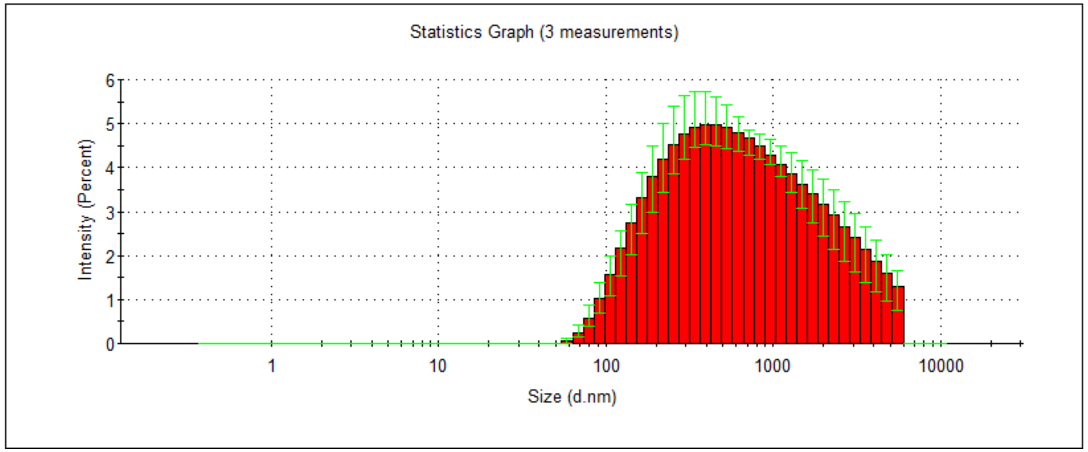

Figure S4: MSZ Nanosuspensions 1.66 mg/mL pH 6.0 (Intensity distribution)

Size d.nm	Mean Number Percent	Std Dev Number Percent
0,4000	0,0	0,0
0,4632	0,0	0,0
0,5365	0,0	0,0
0,6213	0,0	0,0
0,7195	0,0	0,0
0,8332	0,0	0,0
0,9649	0,0	0,0
1,117	0,0	0,0
1,294	0,0	0,0
1,499	0,0	0,0
1,736	0,0	0,0
2,010	0,0	0,0
2,328	0,0	0,0
2,696	0,0	0,0
3,122	0,0	0,0
3,615	0,0	0,0
4,187	0,0	0,0
4,849	0,0	0,0

Size d.nm	Mean Number Percent	Std Dev Number Percent
5,615	0,0	0,0
6,503	0,0	0,0
7,531	0,0	0,0
8,721	0,0	0,0
10,10	0,0	0,0
11,70	0,0	0,0
13,54	0,0	0,0
15,69	0,0	0,0
18,17	0,0	0,0
21,04	0,0	0,0
24,36	0,0	0,0
28,21	0,0	0,0
32,67	0,0	0,0
37,84	0,0	0,0
43,82	0,4	0,6
50,75	3,1	2,9
58,77	9,6	5,6
68,06	16,0	4,3

Size d.nm	Mean Number Percent	Std Dev Number Percent
78,82	17,7	1,5
91,28	15,2	2,3
105,7	11,3	2,6
122,4	7,8	2,2
141,8	5,2	1,7
164,2	3,7	1,3
190,1	2,8	1,0
220,2	2,0	0,7
255,0	1,4	0,5
295,3	1,0	0,4
342,0	0,7	0,3
396,1	0,5	0,2
458,7	0,4	0,1
531,2	0,3	0,1
615,1	0,2	0,1
712,4	0,2	0,1
825,0	0,2	0,1
955,4	0,1	0,0

Size d.nm	Mean Number Percent	Std Dev Number Percent
1106	0,1	0,0
1281	0,1	0,0
1484	0,1	0,0
1718	0,0	0,0
1990	0,0	0,0
2305	0,0	0,0
2669	0,0	0,0
3091	0,0	0,0
3580	0,0	0,0
4145	0,0	0,0
4801	0,0	0,0
5560	0,0	0,0
6439	0,0	0,0
7456	0,0	0,0
8635	0,0	0,0
1,000e4	0,0	0,0

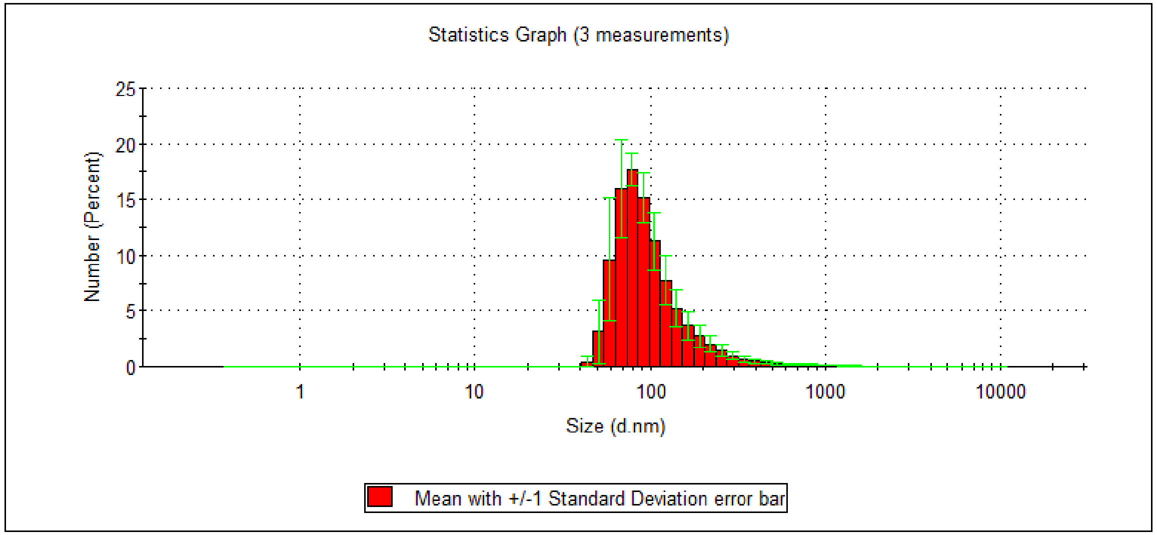

Figure S5: MSZ Nanosuspensions 1.66 mg/mL pH 6.0 (Number distribution)

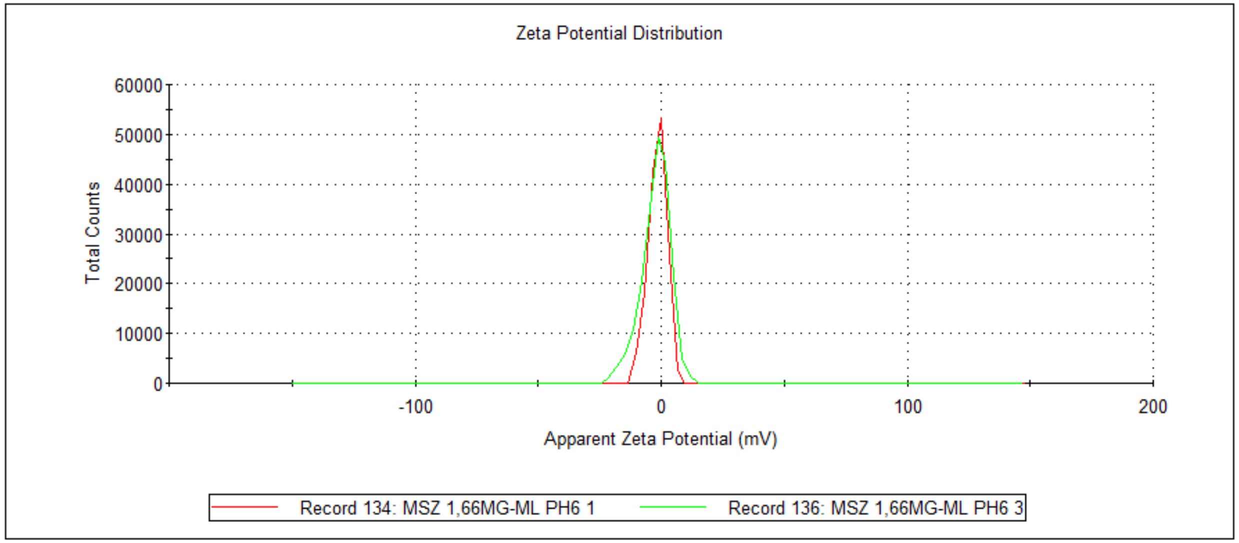

Figure S6: MSZ Nanosuspensions 1.66 mg/mL pH 6.0 (Zeta Potential distribution)

Size d.nm	Mean Intensity Percent	Std Dev Intensity Percent
0,4000	0,0	0,0
0,4632	0,0	0,0
0,5365	0,0	0,0
0,6213	0,0	0,0
0,7195	0,0	0,0
0,8332	0,0	0,0
0,9649	0,0	0,0
1,117	0,0	0,0
1,294	0,0	0,0
1,499	0,0	0,0
1,736	0,0	0,0
2,010	0,0	0,0
2,328	0,0	0,0
2,696	0,0	0,0
3,122	0,0	0,0
3,615	0,0	0,0
4,187	0,0	0,0
4,849	0,0	0,0

Size d.nm	Mean Intensity Percent	Std Dev Intensity Percent
5,615	0,0	0,0
6,503	0,0	0,0
7,531	0,0	0,0
8,721	0,0	0,0
10,10	0,0	0,0
11,70	0,0	0,0
13,54	0,0	0,0
15,69	0,0	0,0
18,17	0,0	0,0
21,04	0,0	0,0
24,36	0,0	0,0
28,21	0,0	0,0
32,67	0,0	0,0
37,84	0,0	0,0
43,82	0,1	0,1
50,75	0,5	0,3
58,77	1,0	0,5
68,06	1,6	0,5

Size d.nm	Mean Intensity Percent	Std Dev Intensity Percent
78,82	1,9	0,5
91,28	2,0	0,5
105,7	1,9	0,6
122,4	1,8	0,5
141,8	1,8	0,4
164,2	2,2	0,3
190,1	2,9	0,6
220,2	3,9	0,9
255,0	5,2	1,2
295,3	6,4	1,2
342,0	7,5	1,1
396,1	8,2	0,9
458,7	8,5	0,5
531,2	8,4	0,3
615,1	7,8	0,6
712,4	6,9	1,0
825,0	5,7	1,2
955,4	4,4	1,3

Size d.nm	Mean Intensity Percent	Std Dev Intensity Percent
1106	3,1	1,3
1281	2,0	1,1
1484	1,1	0,9
1718	0,5	0,6
1990	0,2	0,3
2305	0,1	0,1
2669	0,1	0,2
3091	0,2	0,4
3580	0,3	0,6
4145	0,5	0,8
4801	0,7	0,9
5560	0,8	0,9
6439	0,0	0,0
7456	0,0	0,0
8635	0,0	0,0
1,000e4	0,0	0,0

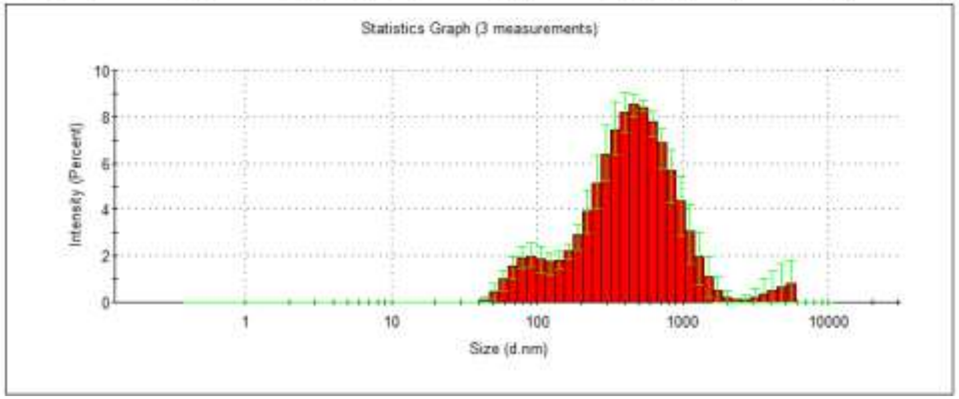

Figure S7: C8:A2-NS MSZ 1.66 mg/mL pH 4.0 (Intensity distribution)

Size d.nm	Mean Number Percent	Std Dev Number Percent	Size d.nm	Mean Number Percent	Std Dev Number Percent	Size d.nm	Mean Number Percent	Std Dev Number Percent	Size d.nm	Mean Number Percent	Std Dev Number Percent
0,4000	0,0	0,0	5,615	0,0	0,0	78,82	11,0	4,6	1106	0,0	0,0
0,4632	0,0	0,0	6,503	0,0	0,0	91,28	5,7	3,2	1281	0,0	0,0
0,5365	0,0	0,0	7,531	0,0	0,0	105,7	2,8	1,9	1484	0,0	0,0
0,6213	0,0	0,0	8,721	0,0	0,0	122,4	1,3	1,0	1718	0,0	0,0
0,7195	0,0	0,0	10,10	0,0	0,0	141,8	0,7	0,6	1990	0,0	0,0
0,8332	0,0	0,0	11,70	0,0	0,0	164,2	0,5	0,4	2305	0,0	0,0
0,9649	0,0	0,0	13,54	0,0	0,0	190,1	0,4	0,3	2669	0,0	0,0
1,117	0,0	0,0	15,69	0,0	0,0	220,2	0,3	0,2	3091	0,0	0,0
1,294	0,0	0,0	18,17	0,0	0,0	255,0	0,3	0,2	3580	0,0	0,0
1,499	0,0	0,0	21,04	0,0	0,0	295,3	0,2	0,1	4145	0,0	0,0
1,736	0,0	0,0	24,36	0,0	0,0	342,0	0,2	0,1	4801	0,0	0,0
2,010	0,0	0,0	28,21	0,0	0,0	396,1	0,1	0,1	5560	0,0	0,0
2,328	0,0	0,0	32,67	0,0	0,0	458,7	0,1	0,1	6439	0,0	0,0
2,696	0,0	0,0	37,84	2,7	2,9	531,2	0,1	0,1	7456	0,0	0,0
3,122	0,0	0,0	43,82	11,4	7,8	615,1	0,1	0,0	8635	0,0	0,0
3,615	0,0	0,0	50,75	21,0	6,6	712,4	0,0	0,0	1,000e4	0,0	0,0
4,187	0,0	0,0	58,77	23,2	1,2	825,0	0,0	0,0			
4,849	0,0	0,0	68,06	18,0	4,4	955,4	0,0	0,0			

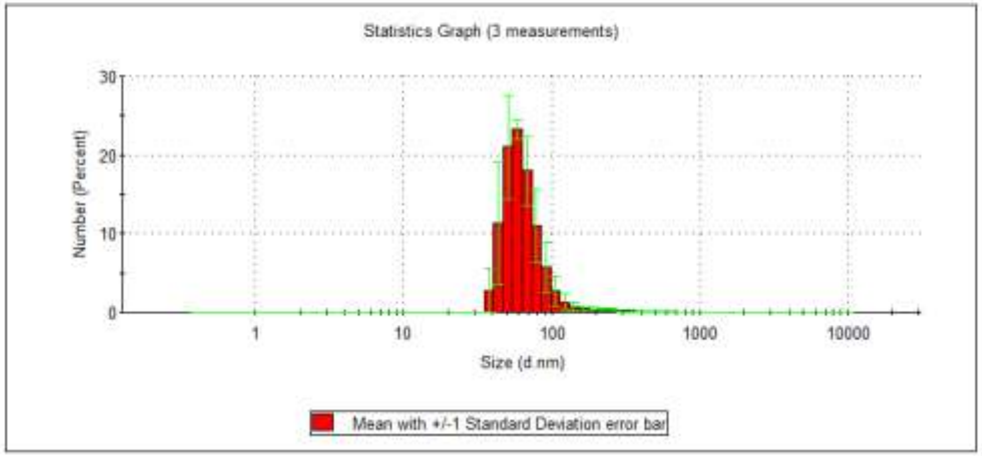

Figure S8: C8:A2-NS MSZ 1.66 mg/mL pH 4.0 (Number distribution)

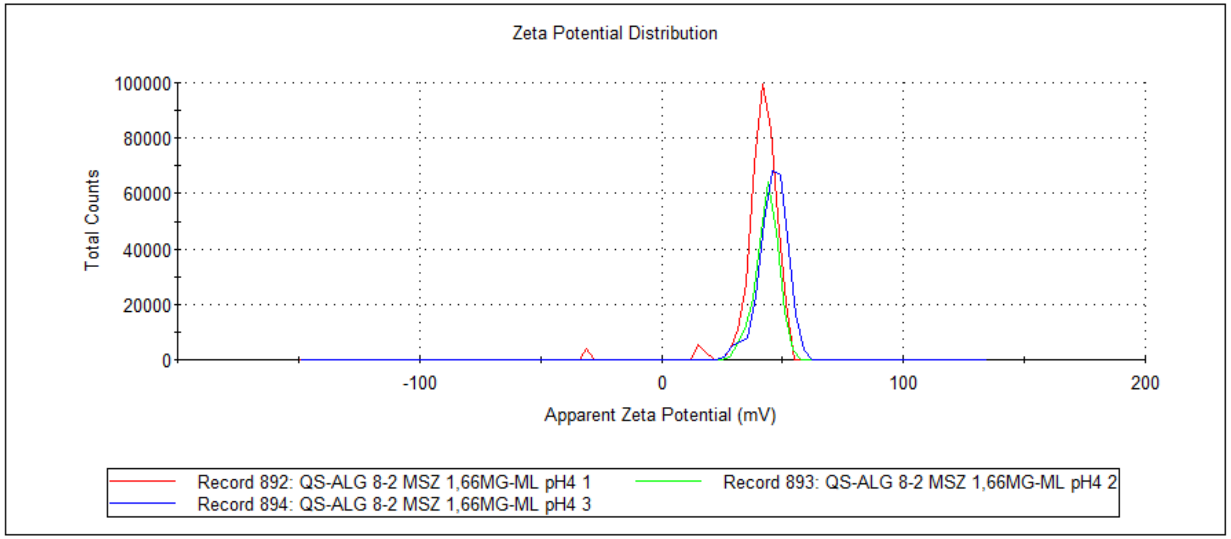

Figure S9: C8:A2-NS MSZ 1.66 mg/mL pH 4.0 (Zeta Potential distribution)

Size d.nm	Mean Intensity Percent	Std Dev Intensity Percent	Size d.nm	Mean Intensity Percent	Std Dev Intensity Percent	Size d.nm	Mean Intensity Percent	Std Dev Intensity Percent	Size d.nm	Mean Intensity Percent	Std Dev Intensity Percent
0,4000	0,0	0,0	5,615	0,0	0,0	78,82	1,1	1,0	1106	3,9	1,9
0,4632	0,0	0,0	6,503	0,0	0,0	91,28	1,4	1,5	1281	2,7	1,5
0,5365	0,0	0,0	7,531	0,0	0,0	105,7	1,5	1,6	1484	1,6	1,0
0,6213	0,0	0,0	8,721	0,0	0,0	122,4	1,8	1,4	1718	0,8	0,6
0,7195	0,0	0,0	10,10	0,0	0,0	141,8	2,0	0,7	1990	0,3	0,2
0,8332	0,0	0,0	11,70	0,0	0,0	164,2	2,5	0,2	2305	0,1	0,1
0,9649	0,0	0,0	13,54	0,0	0,0	190,1	3,1	1,1	2669	0,2	0,3
1,117	0,0	0,0	15,69	0,0	0,0	220,2	3,9	1,9	3091	0,3	0,5
1,294	0,0	0,0	18,17	0,0	0,0	255,0	4,8	2,4	3580	0,4	0,7
1,499	0,0	0,0	21,04	0,0	0,1	295,3	5,8	2,4	4145	0,5	0,9
1,736	0,0	0,0	24,36	0,1	0,2	342,0	6,7	2,1	4801	0,6	1,0
2,010	0,0	0,0	28,21	0,2	0,2	396,1	7,4	1,5	5560	0,6	1,0
2,328	0,0	0,0	32,67	0,3	0,3	458,7	7,9	0,6	6439	0,0	0,0
2,696	0,0	0,0	37,84	0,4	0,4	531,2	8,0	0,3	7456	0,0	0,0
3,122	0,0	0,0	43,82	0,5	0,6	615,1	7,8	1,1	8635	0,0	0,0
3,615	0,0	0,0	50,75	0,6	0,6	712,4	7,2	1,7	1,000e4	0,0	0,0
4,187	0,0	0,0	58,77	0,7	0,3	825,0	6,3	2,1			
4,849	0,0	0,0	68,06	0,9	0,3	955,4	5,2	2,1			

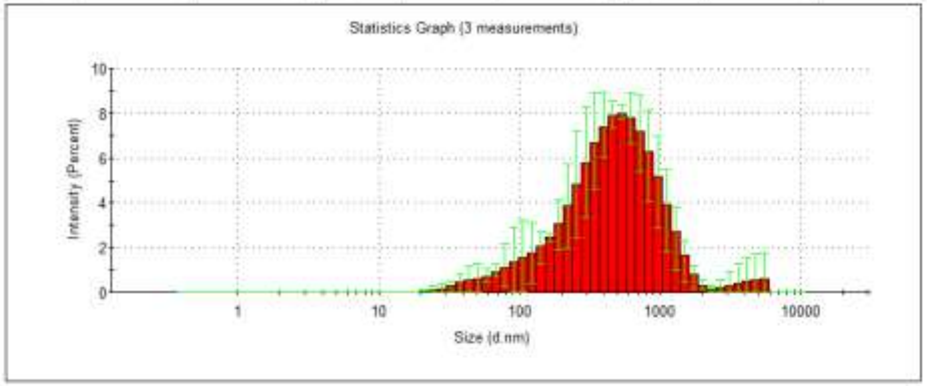

Figure S10: C8:A2-NS MSZ 1.66 mg/mL pH 6.0 (Intensity distribution)

Size d.nm	Mean Number Percent	Std Dev Number Percent
0,4000	0,0	0,0
0,4632	0,0	0,0
0,5365	0,0	0,0
0,6213	0,0	0,0
0,7195	0,0	0,0
0,8332	0,0	0,0
0,9649	0,0	0,0
1,117	0,0	0,0
1,294	0,0	0,0
1,499	0,0	0,0
1,736	0,0	0,0
2,010	0,0	0,0
2,328	0,0	0,0
2,696	0,0	0,0
3,122	0,0	0,0
3,615	0,0	0,0
4,187	0,0	0,0
4,849	0,0	0,0

Size d.nm	Mean Number Percent	Std Dev Number Percent
5,615	0,0	0,0
6,503	0,0	0,0
7,531	0,0	0,0
8,721	0,0	0,0
10,10	0,0	0,0
11,70	0,0	0,0
13,54	0,0	0,0
15,69	0,0	0,1
18,17	2,7	4,7
21,04	8,0	13,9
24,36	10,8	15,7
28,21	11,6	10,4
32,67	12,0	12,9
37,84	10,0	13,5
43,82	6,3	9,3
50,75	5,0	3,9
58,77	7,4	9,6
68,06	8,9	14,3

Size d.nm	Mean Number Percent	Std Dev Number Percent
78,82	7,2	12,2
91,28	4,6	7,9
105,7	2,5	4,3
122,4	1,2	2,1
141,8	0,6	1,0
164,2	0,3	0,4
190,1	0,2	0,2
220,2	0,1	0,1
255,0	0,1	0,1
295,3	0,1	0,1
342,0	0,1	0,1
396,1	0,1	0,1
458,7	0,1	0,1
531,2	0,0	0,1
615,1	0,0	0,1
712,4	0,0	0,1
825,0	0,0	0,0
955,4	0,0	0,0

Size d.nm	Mean Number Percent	Std Dev Number Percent
1106	0,0	0,0
1281	0,0	0,0
1484	0,0	0,0
1718	0,0	0,0
1990	0,0	0,0
2305	0,0	0,0
2669	0,0	0,0
3091	0,0	0,0
3580	0,0	0,0
4145	0,0	0,0
4801	0,0	0,0
5560	0,0	0,0
6439	0,0	0,0
7456	0,0	0,0
8635	0,0	0,0
1,000e4	0,0	0,0

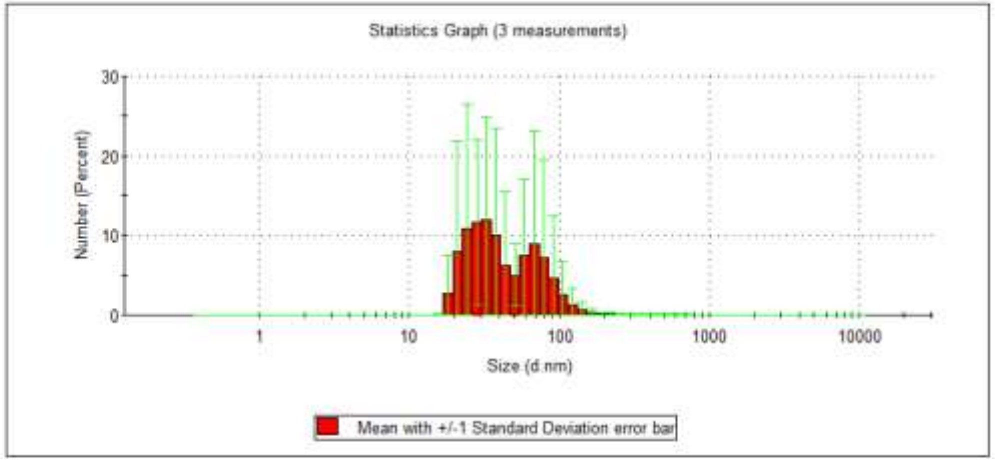

Figure S11: C8:A2-NS MSZ 1.66 mg/mL pH 6.0 (Number distribution)

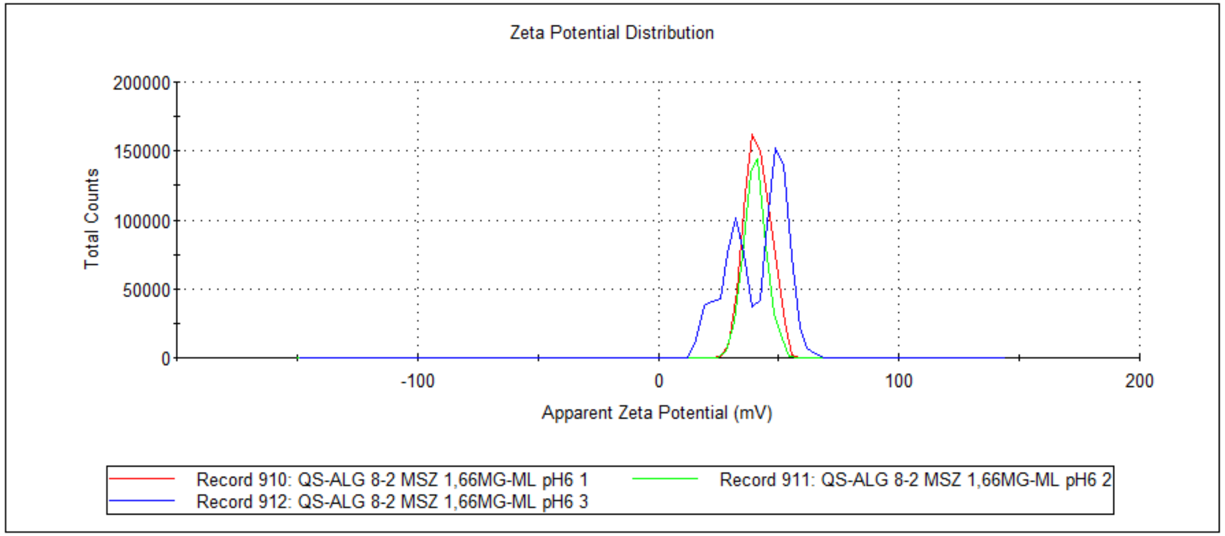

Figure S12: C8:A2-NS MSZ 1.66 mg/mL pH 6.0 (Zeta Potential distribution)

Size d.nm	Mean Intensity Percent	Std Dev Intensity Percent
0,4000	0,0	0,0
0,4632	0,0	0,0
0,5365	0,0	0,0
0,6213	0,0	0,0
0,7195	0,0	0,0
0,8332	0,0	0,0
0,9649	0,0	0,0
1,117	0,0	0,0
1,294	0,0	0,0
1,499	0,0	0,0
1,736	0,0	0,0
2,010	0,0	0,0
2,328	0,0	0,0
2,696	0,0	0,0
3,122	0,0	0,0
3,615	0,0	0,0
4,187	0,0	0,0
4,849	0,0	0,0

Size d.nm	Mean Intensity Percent	Std Dev Intensity Percent
5,615	0,0	0,0
6,503	0,0	0,0
7,531	0,0	0,0
8,721	0,0	0,0
10,10	0,0	0,0
11,70	0,0	0,0
13,54	0,0	0,0
15,69	0,0	0,0
18,17	0,0	0,0
21,04	0,0	0,0
24,36	0,1	0,2
28,21	0,3	0,4
32,67	0,4	0,6
37,84	0,6	0,6
43,82	0,6	0,5
50,75	0,6	0,4
58,77	0,6	0,4
68,06	0,6	0,5

Size d.nm	Mean Intensity Percent	Std Dev Intensity Percent
78,82	0,6	0,6
91,28	0,6	0,6
105,7	0,8	0,5
122,4	1,1	0,3
141,8	1,8	0,2
164,2	2,8	0,4
190,1	3,9	0,6
220,2	5,2	0,6
255,0	6,4	0,4
295,3	7,3	0,2
342,0	7,9	0,4
396,1	7,9	0,7
458,7	7,6	0,9
531,2	6,7	1,1
615,1	5,6	1,1
712,4	4,3	1,0
825,0	3,0	0,9
955,4	1,9	0,8

Size d.nm	Mean Intensity Percent	Std Dev Intensity Percent
1106	1,0	0,6
1281	0,5	0,5
1484	0,4	0,4
1718	0,4	0,6
1990	0,5	0,8
2305	0,7	1,0
2669	1,2	1,0
3091	1,9	0,8
3580	2,7	0,6
4145	3,4	0,7
4801	4,0	1,2
5560	4,2	1,6
6439	0,0	0,0
7456	0,0	0,0
8635	0,0	0,0
1,000e4	0,0	0,0

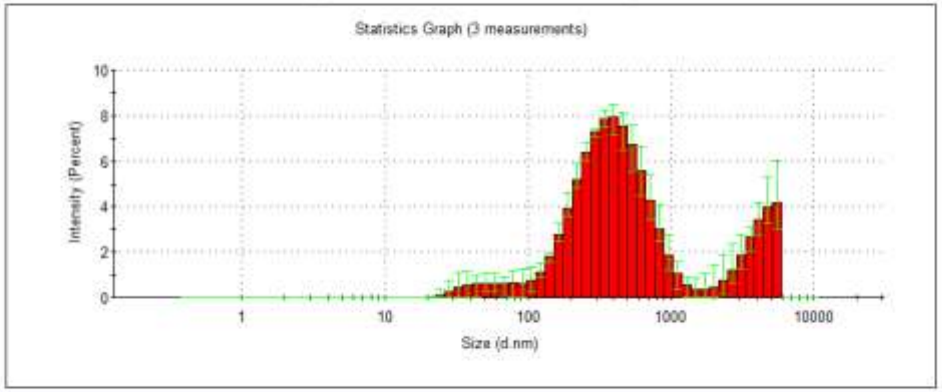

Figure S13: C8:A1:H1-NS MSZ 1.66 mg/mL pH 4.0 (Intensity distribution)

Size d.nm	Mean Number Percent	Std Dev Number Percent
0,4000	0,0	0,0
0,4632	0,0	0,0
0,5365	0,0	0,0
0,6213	0,0	0,0
0,7195	0,0	0,0
0,8332	0,0	0,0
0,9649	0,0	0,0
1,117	0,0	0,0
1,294	0,0	0,0
1,499	0,0	0,0
1,736	0,0	0,0
2,010	0,0	0,0
2,328	0,0	0,0
2,696	0,0	0,0
3,122	0,0	0,0
3,615	0,0	0,0
4,187	0,0	0,0
4,849	0,0	0,0

Size d.nm	Mean Number Percent	Std Dev Number Percent
5,615	0,0	0,0
6,503	0,0	0,0
7,531	0,0	0,0
8,721	0,0	0,0
10,10	0,0	0,0
11,70	0,0	0,0
13,54	0,0	0,0
15,69	0,0	0,0
18,17	0,8	1,4
21,04	4,3	7,4
24,36	8,9	14,7
28,21	12,0	13,8
32,67	13,6	12,0
37,84	12,4	13,3
43,82	10,8	8,5
50,75	11,1	9,9
58,77	10,0	12,8
68,06	6,9	10,1

Size d.nm	Mean Number Percent	Std Dev Number Percent
78,82	3,9	6,1
91,28	2,0	3,2
105,7	1,0	1,6
122,4	0,5	0,8
141,8	0,3	0,5
164,2	0,3	0,4
190,1	0,2	0,4
220,2	0,2	0,3
255,0	0,2	0,3
295,3	0,1	0,2
342,0	0,1	0,2
396,1	0,1	0,1
458,7	0,1	0,1
531,2	0,0	0,1
615,1	0,0	0,0
712,4	0,0	0,0
825,0	0,0	0,0
955,4	0,0	0,0

Size d.nm	Mean Number Percent	Std Dev Number Percent
1106	0,0	0,0
1281	0,0	0,0
1484	0,0	0,0
1718	0,0	0,0
1990	0,0	0,0
2305	0,0	0,0
2669	0,0	0,0
3091	0,0	0,0
3580	0,0	0,0
4145	0,0	0,0
4801	0,0	0,0
5560	0,0	0,0
6439	0,0	0,0
7456	0,0	0,0
8635	0,0	0,0
1,000e4	0,0	0,0

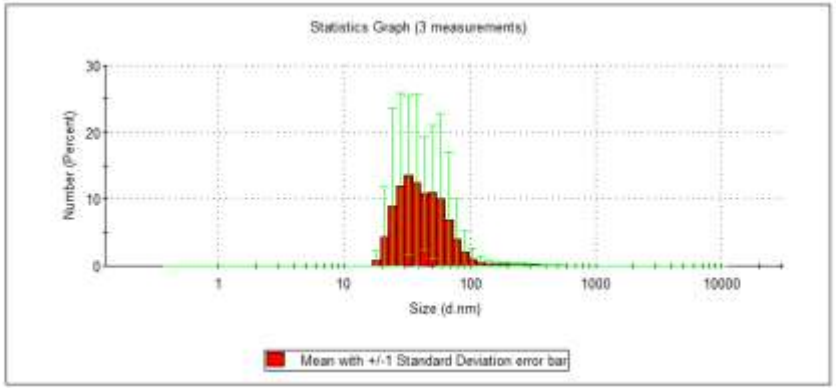

Figure S14: C8:A1:H1-NS MSZ 1.66 mg/mL pH 4.0 (Number distribution)

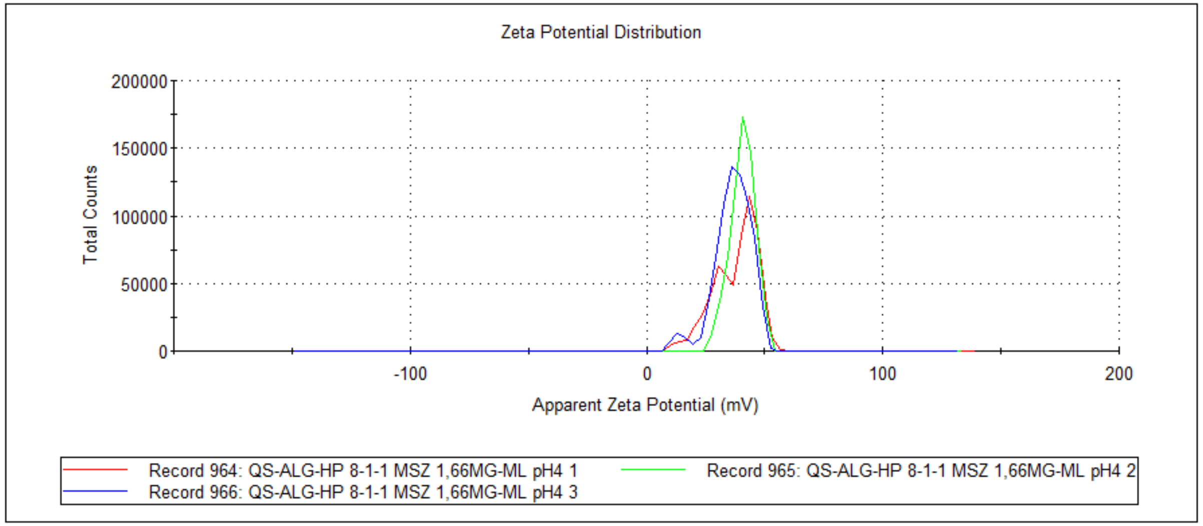

Figure S15: C8:A1-H1-NS MSZ 1.66 mg/mL pH 4.0 (Zeta Potential distribution)

Size d.nm	Mean Intensity Percent	Std Dev Intensity Percent
0,4000	0,0	0,0
0,4632	0,0	0,0
0,5365	0,0	0,0
0,6213	0,0	0,0
0,7195	0,0	0,0
0,8332	0,0	0,0
0,9649	0,0	0,0
1,117	0,0	0,0
1,294	0,0	0,0
1,499	0,0	0,0
1,736	0,0	0,0
2,010	0,0	0,0
2,328	0,0	0,0
2,696	0,0	0,0
3,122	0,0	0,0
3,615	0,0	0,0
4,187	0,0	0,0
4,849	0,0	0,0

Size d.nm	Mean Intensity Percent	Std Dev Intensity Percent
5,615	0,0	0,0
6,503	0,0	0,0
7,531	0,0	0,0
8,721	0,0	0,0
10,10	0,0	0,0
11,70	0,0	0,0
13,54	0,1	0,1
15,69	0,2	0,1
18,17	0,2	0,1
21,04	0,2	0,1
24,36	0,2	0,0
28,21	0,3	0,1
32,67	0,3	0,2
37,84	0,4	0,3
43,82	0,6	0,3
50,75	0,7	0,3
58,77	0,8	0,2
68,06	1,0	0,1

Size d.nm	Mean Intensity Percent	Std Dev Intensity Percent
78,82	1,2	0,3
91,28	1,4	0,5
105,7	1,7	0,6
122,4	2,2	0,6
141,8	2,7	0,4
164,2	3,3	0,2
190,1	4,0	0,3
220,2	4,6	0,7
255,0	5,1	1,1
295,3	5,6	1,4
342,0	5,9	1,6
396,1	6,0	1,6
458,7	5,9	1,4
531,2	5,7	1,2
615,1	5,3	0,8
712,4	4,8	0,4
825,0	4,3	0,2
955,4	3,8	0,6

Size d.nm	Mean Intensity Percent	Std Dev Intensity Percent
1106	3,2	0,9
1281	2,7	1,2
1484	2,3	1,3
1718	1,9	1,4
1990	1,7	1,3
2305	1,5	1,2
2669	1,4	1,0
3091	1,4	0,7
3580	1,4	0,4
4145	1,4	0,2
4801	1,3	0,1
5560	1,3	0,3
6439	0,0	0,0
7456	0,0	0,0
8635	0,0	0,0
1,000e4	0,0	0,0

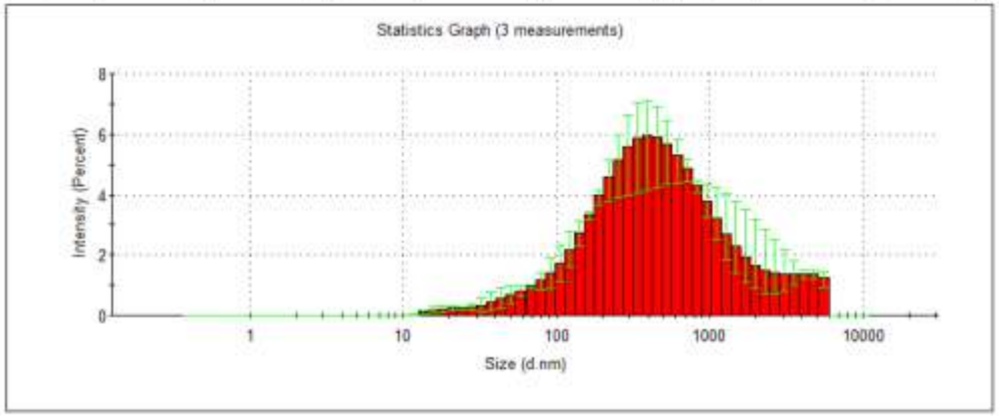

Figure S16: C8:A1:H1-NS MSZ 1.66 mg/mL pH 6.0 (Intensity distribution)

Size d.nm	Mean Number Percent	Std Dev Number Percent
0,4000	0,0	0,0
0,4632	0,0	0,0
0,5365	0,0	0,0
0,6213	0,0	0,0
0,7195	0,0	0,0
0,8332	0,0	0,0
0,9649	0,0	0,0
1,117	0,0	0,0
1,294	0,0	0,0
1,499	0,0	0,0
1,736	0,0	0,0
2,010	0,0	0,0
2,328	0,0	0,0
2,696	0,0	0,0
3,122	0,0	0,0
3,615	0,0	0,0
4,187	0,0	0,0
4,849	0,0	0,0

Size d.nm	Mean Number Percent	Std Dev Number Percent
5,615	0,0	0,0
6,503	0,0	0,0
7,531	0,0	0,0
8,721	0,9	1,6
10,10	8,1	4,1
11,70	21,1	3,2
13,54	26,3	1,9
15,69	20,1	3,1
18,17	11,5	2,1
21,04	5,8	1,6
24,36	2,9	1,7
28,21	1,5	1,4
32,67	0,8	1,0
37,84	0,5	0,6
43,82	0,2	0,3
50,75	0,1	0,1
58,77	0,1	0,1
68,06	0,0	0,0

Size d.nm	Mean Number Percent	Std Dev Number Percent
78,82	0,0	0,0
91,28	0,0	0,0
105,7	0,0	0,0
122,4	0,0	0,0
141,8	0,0	0,0
164,2	0,0	0,0
190,1	0,0	0,0
220,2	0,0	0,0
255,0	0,0	0,0
295,3	0,0	0,0
342,0	0,0	0,0
396,1	0,0	0,0
458,7	0,0	0,0
531,2	0,0	0,0
615,1	0,0	0,0
712,4	0,0	0,0
825,0	0,0	0,0
955,4	0,0	0,0

Size d.nm	Mean Number Percent	Std Dev Number Percent
1106	0,0	0,0
1281	0,0	0,0
1484	0,0	0,0
1718	0,0	0,0
1990	0,0	0,0
2305	0,0	0,0
2669	0,0	0,0
3091	0,0	0,0
3580	0,0	0,0
4145	0,0	0,0
4801	0,0	0,0
5560	0,0	0,0
6439	0,0	0,0
7456	0,0	0,0
8635	0,0	0,0
1,000e4	0,0	0,0

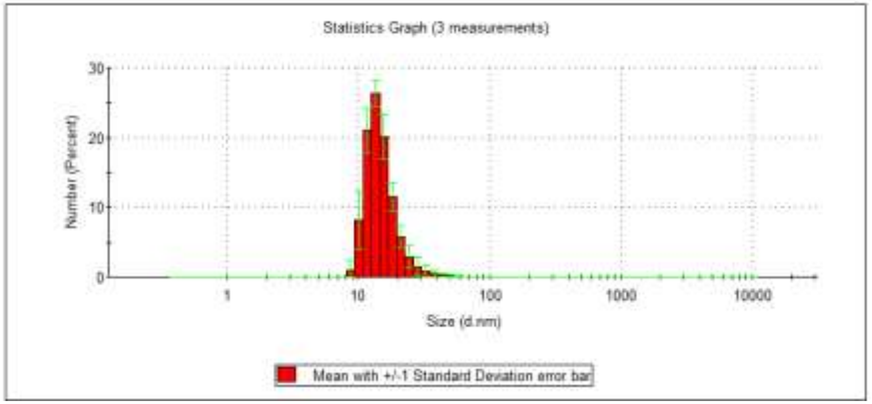

Figure S17: C8:A1:H1-NS MSZ 1.66 mg/mL pH 6.0 (Number distribution)

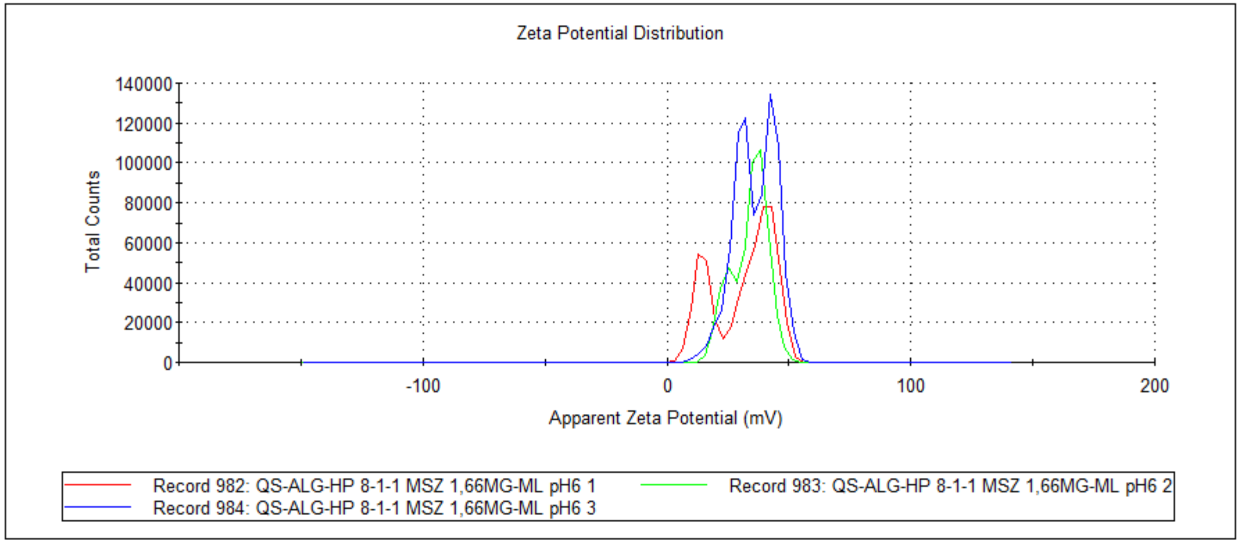

Figure S18: C8:A1-H1-NS MSZ 1.66 mg/mL pH 6.0 (Zeta Potential distribution)
